# A global survey of intramolecular isopeptide bonds

**DOI:** 10.1002/pro.70342

**Published:** 2025-11-14

**Authors:** Francesco Costa, Ioannis Riziotis, Antonina Andreeva, Delhi Kalwan, Jennifer de Jong, Philip Hinchliffe, Fabio Parmeggiani, Paul R. Race, Steven G. Burston, Alex Bateman, Rob Barringer

**Affiliations:** ^1^ European Molecular Biology Laboratory European Bioinformatics Institute (EMBL‐EBI) Hinxton UK; ^2^ School of Biochemistry University of Bristol Bristol UK; ^3^ School of Cellular and Molecular Medicine University of Bristol Bristol UK; ^4^ School of Chemistry University of Bristol Bristol UK; ^5^ School of Pharmacy and Pharmaceutical Sciences Cardiff University Cardiff UK; ^6^ School of Natural and Environmental Sciences, Devonshire Building Newcastle University Newcastle upon Tyne UK

**Keywords:** adhesion, biofilm, fibrillar adhesin, host‐pathogen, isopeptide, pilus

## Abstract

Many proteins harbor covalent intramolecular bonds that enhance their stability and resistance to thermal, mechanical, and proteolytic insults. Intramolecular isopeptide bonds represent one such covalent interaction, yet their distribution across protein domains and organisms has been largely unexplored. Here, we sought to address this by employing a large‐scale prediction of intramolecular isopeptide bonds in the AlphaFold database using the structural template‐based software Isopeptor. Our findings reveal an extensive phyletic distribution in bacterial and archaeal surface proteins resembling fibrillar adhesins and pilins. All identified intramolecular isopeptide bonds are found in two structurally distinct folds, CnaA‐like or CnaB‐like, from a relatively small set of related Pfam families, including 10 novel families that we predict to contain intramolecular isopeptide bonds. One CnaA‐like domain of unknown function, DUF11 (renamed here to “CLIPPER”) is broadly distributed in cell‐surface proteins from Gram‐positive bacteria, Gram‐negative bacteria, and archaea, and is structurally and biophysically characterized in this work. Using x‐ray crystallography, we resolve a CLIPPER domain from a Gram‐negative fibrillar adhesin that contains an intramolecular isopeptide bond and further demonstrate that it imparts thermostability and resistance to proteolysis. Our findings demonstrate the extensive distribution of intramolecular isopeptide bond‐containing protein domains in nature and structurally resolve the previously cryptic CLIPPER domain.

## 1 INTRODUCTION

The isopeptide bond is a class of covalent amide bond that forms between the amino and carboxamide/carboxyl groups of two side chains of a polypeptide or between a side chain and a terminus. In nature, isopeptide bonds enable either the cross‐linking of two points within a single polypeptide chain (intramolecular isopeptide bonds) or between two points of two different polypeptide chains (intermolecular isopeptide bonds). Enzyme‐catalyzed intermolecular isopeptide bonds play a role in many biological processes (Kang & Baker, 2011), including protein ubiquitination, where the target protein is covalently tethered to the ubiquitin protein (Hershko & Ciechanover, 1998); the coagulation pathway, where Factor XIII catalyzes crosslinking of fibrin (Muszbek et al., 1996); and in cell‐wall anchored surface proteins of Gram‐positive bacteria, whereby pilins are crosslinked to the pentaglycine peptidoglycan bridge at the microbial cell surface (Hendrickx et al., 2011). Notably, some intermolecular isopeptide bonds form through an autocatalytic mechanism, such as between capsid subunits of various bacteriophages (Helgstrand et al., 2003; Podgorski et al., 2023), where the isopeptide bonds form within a hydrophobic pocket between lysine and asparagine side chains, and are catalyzed by a nearby glutamate side chain (Tso et al., 2017).

**FIGURE 1:**
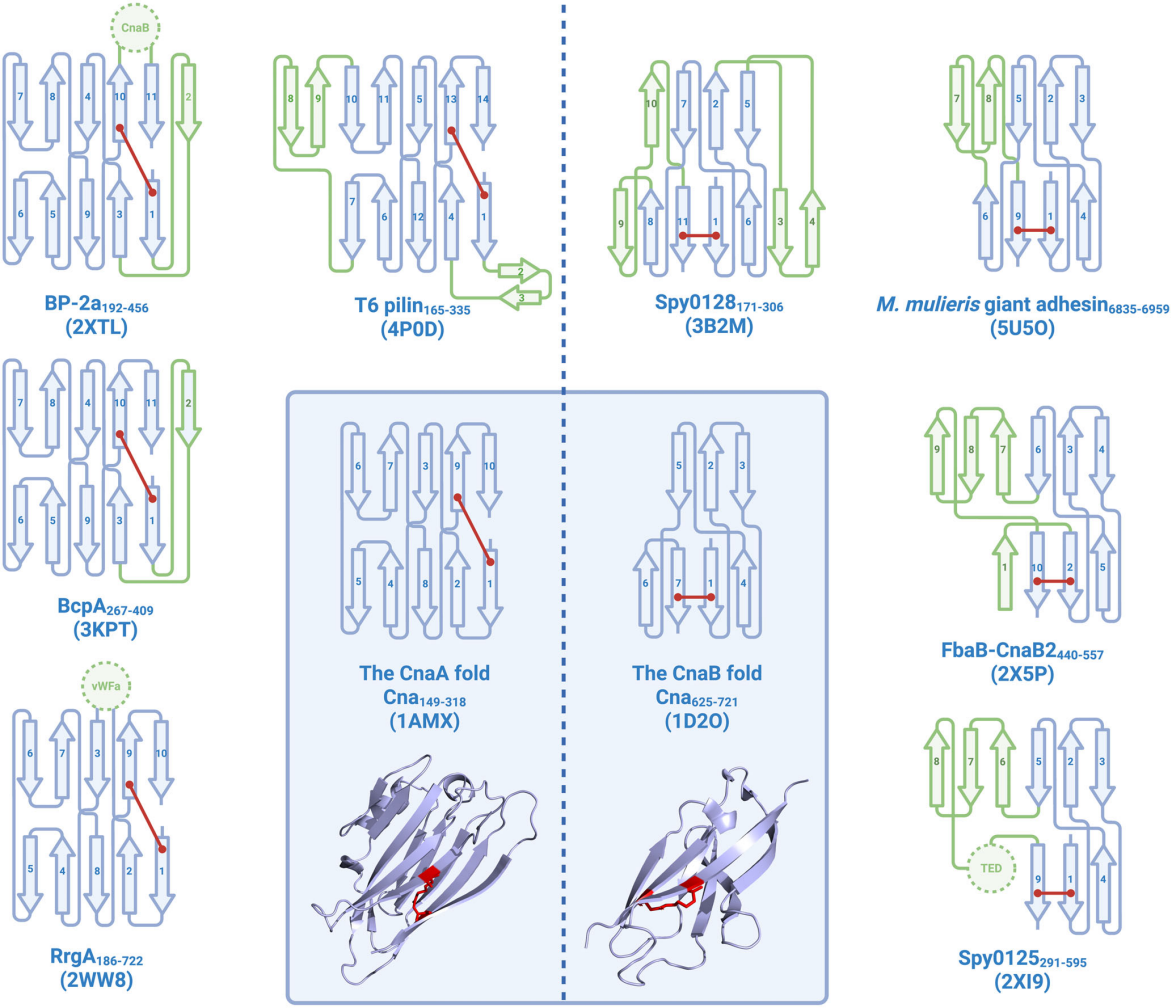
β‐strand arrangement in CnaA and CnaB folds. Isopeptide bonds are indicated by red lines, along with cartoon depictions of the folds. Alternative CnaA/CnaB topologies are depicted in the periphery. Differences relative to archetypal CnaA/CnaB folds are highlighted in green. Domain inserts are depicted as dotted circles and labeled as follows: CnaB, CnaB‐like fold; vWFa, von Willebrand factor type A domain; TED, thioester domain.

Over the past two decades, a second subclass of autocatalytic isopeptide bond has been investigated: the intramolecular isopeptide bond. The first intramolecular isopeptide bond to be structurally resolved was by Kang et al. (2007), housed within the hydrophobic core of a β‐sandwich domain of the bacterial pilin Spy0128. This isopeptide bond was formed between the lysine and asparagine (Lys‐Asn) side chains of adjacent β‐strands, catalyzed by a proximal glutamate side chain (Kang et al., 2007). Subsequently, several other intramolecular isopeptide bond domains (IPDs) have been identified, invariably formed between side chains within the core of β‐sandwich folds. These folds can be grouped into two distinct structural families sharing a Greek‐key motif: CnaA‐like domains (an Ig‐like fold that typically forms Lys‐Asn cross‐links catalyzed by an aspartate) and CnaB‐like domains (a transthyretin‐like fold that typically forms Lys‐Asn or Lys‐Asp cross‐links catalyzed by a glutamate; Kang & Baker, 2009; Kang & Baker, 2012). In CnaA‐like domains, intramolecular isopeptide bond‐forming residues are located on opposing β‐sheets between the first and penultimate β‐strands, while in CnaB‐like domains they are positioned on the same β‐sheet, between adjacent first and last β‐strands. Interestingly, both folds are tolerant of domain insertions within loop regions, usually of intramolecular isopeptide or adhesion domains (Izoré et al., 2010; Pointon et al., 2010; Spraggon et al., 2010; Figure 1).

Functionally, intramolecular isopeptide bonds are thought to enable resistance to various stresses at the cell surface. In 2007, Kang et al. demonstrated that intramolecular isopeptide bonds bestow increased proteolytic resistance and thermostability to the CnaB‐like domains of Spy0128, which later proved characteristic of CnaA‐like and CnaB‐like domains more broadly (Chaurasia et al., 2016; El Mortaji et al., 2012; Hagan et al., 2010; Heidler et al., 2021; Hendrickx et al., 2012; Kang et al., 2007; Kang & Baker, 2009; Kang & Baker, 2011; Zähner et al., 2011). Further work revealed that the intramolecular isopeptide bonds of CnaB‐like domains in Spy0128 enable resistance to mechanical unfolding, with these polypeptides proving inextensible under atomic force microscopy (AFM)‐induced tension (Alegre‐Cebollada et al., 2010). In contrast, CnaA‐like folds were found to unfold partially under tension, leading to the molecular “shock absorber” hypothesis whereby CnaA‐like domains enable adherence under shear forces by dissipating mechanical perturbations (Echelman et al., 2016).

To date, intramolecular isopeptide bonds have primarily been identified in adhesive pili and fibrillar adhesins of Gram‐positive bacteria, leading some to question whether they may also be found in Gram‐negative bacteria, archaea, eukaryotes, or viruses (Schwarz‐Linek & Banfield, 2014). This open question was partially answered in 2021, when an intramolecular isopeptide bond was identified in a pilin of a Gram‐negative bacterium (Heidler et al., 2021). Despite this finding, the prevalence and phyletic distribution of intramolecular IPDs in nature have not been systematically probed, and the proteins harboring these domains have not been systematically characterized.

Here, we present the first large‐scale prediction of naturally occurring intramolecular isopeptide bonds in the AlphaFold Database, providing insights into their distribution across organisms and protein domains. Using x‐ray crystallography, we subsequently resolve an intramolecular isopeptide bond in DUF11, a domain of unknown function found in fibrillar adhesins of Gram‐positive bacteria, Gram‐negative bacteria, and archaea, and confirm that the isopeptide bond imparts significant thermostability and proteolytic resistance. Our work reveals that intramolecular isopeptide bonds frequently appear in stalks of bacterial and archaeal fibrillar adhesins and likely facilitate adherence under various stressful conditions.

## 2 RESULTS

### 2.1 Characteristics of intramolecular isopeptide bonds

Previous experimental and computational studies revealed that hydrophobic environments likely facilitate intramolecular isopeptide bond formation (Hagan et al., 2010; Hu et al., 2011). However, a comprehensive analysis of the environment of intramolecular isopeptide bonds has not yet been undertaken. Consequently, we first quantified the solvent accessibility of intramolecular isopeptide bonds within protein structures deposited in the PDB and characterized the physicochemical properties of the environment surrounding the bond.

**FIGURE 2:**
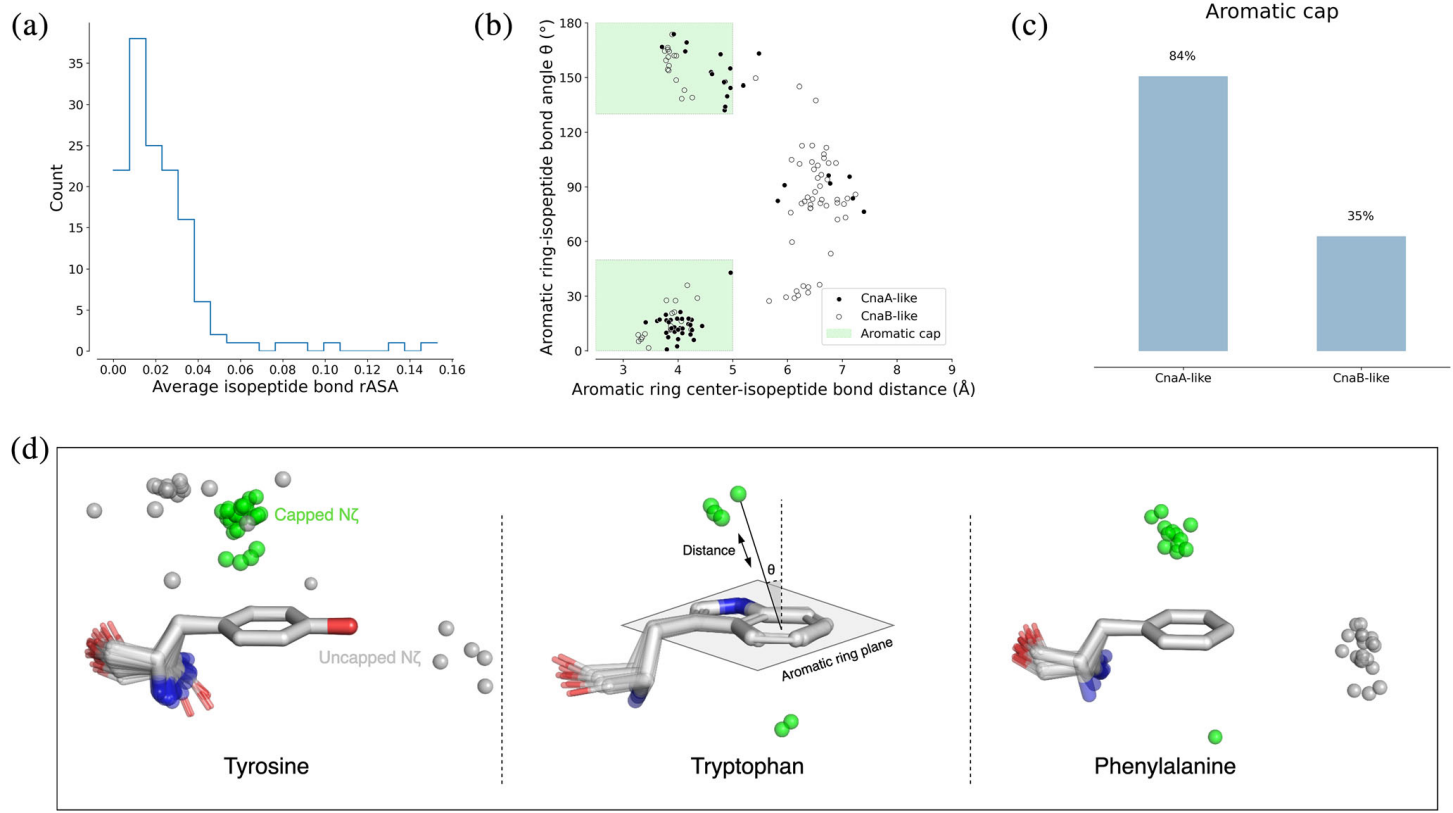
(a) Relative accessible solvent area (rASA) averaged across the three isopeptide bond residues. (b) Scatter plot displaying distance and angles between the intramolecular isopeptide bond and aromatic ring plane (distance calculated between the lysine Nζ atom and the centroid of the aromatic ring, angle calculated between the lysine Nζ atom‐centroid vector and the normal of the aromatic ring plane). (c) Percentage of intramolecular isopeptide bonds with an aromatic cap, defined as an isopeptide bond within 5 Å of the aromatic ring plane, at an angle <50**°** or >130**°** from the normal of the aromatic ring plane (see panel b). Only one PDB entry was assessed per sequence‐identical domain. (d) Positioning of Nζ isopeptide bond atoms around their proximal aromatic ring. “Capped Nζ” refers to isopeptide bonds demonstrating an aromatic cap relationship with a proximal aromatic ring. Panels (a), (b), and (d) include redundant proteins.

Using a PDB dataset of all known intramolecular isopeptide structures collated in prior work (Costa et al., 2025), we found that the relative solvent accessible surface area (rASA) of most intramolecular isopeptide bonds was <0.05, confirming the buried nature of isopeptide bonds within hydrophobic cores (Kang et al., 2007) (Figure 2a). When assessing *cis*/*trans* conformations of intramolecular isopeptide bonds, we found that no Lys‐Asp bonds are present in the *cis* conformation, while Lys‐Asn bonds are equally found in either *cis* (50%) or *trans* (48%) conformations (with 2% found in intermediate conformations). This builds on our previous observation that 67% of CnaB‐like domains favor the *cis* conformation while 73% of CnaA‐like domains favor the *trans* conformation (Costa et al., 2025).

To characterize the local environment, we mapped residues within 6 Å of intramolecular isopeptide bonds (Figure S1A). We observed that the surrounding amino acid distributions are similar between CnaA and CnaB‐like folds, largely consisting of hydrophobic amino acids and aromatic residues (a feature which has only been qualitatively observed to date, Kang et al., 2007; Kang & Baker, 2011). We noted that the Nζ atom of the isopeptide bond is often located ≤5 Å above the plane of an aromatic sidechain, with the isopeptide bond Nζ atom ±50° from the normal of the aromatic ring plane. This arrangement resembles “above ring” amino‐aromatic interactions (Figure 2b,d; Singh & Thornton, 1990; Mitchell et al., 1994). We describe aromatic side chains positioned in such a way as “aromatic caps,” which appear more prevalent in CnaA‐like domains (Figure 2c) but are notably absent in folds harboring insertions of large domains between isopeptide bond‐contributing residues (e.g., in the CnaA‐like domain of the RrgA pilin and the CnaB‐like domain of the Spy0125 pilin; Izoré et al., 2010; Pointon et al., 2010). Aromatic caps are more prevalent in *cis* Lys‐Asn intramolecular isopeptide bonds (94% vs. 24% prevalence in *cis*/*trans* conformations, respectively), and are absent in all PDB structures containing Lys‐Asp bonds. Further analysis found that a consistent portion of aromatic caps engage in stacking interactions with proximal isopeptide bonds, while H‐bond interactions are rare (again reminiscent of amino‐aromatic interactions; Mitchell et al., 1994; Figure S1B). Our analyses also frequently revealed the presence of electron density consistent with a water or ammonia molecule within 5 Å of the intramolecular isopeptide bond oxygen in 54% of CnaB‐like and 59% of CnaA‐like domains (Table S1), usually buried in the domain core.

### 2.2 Large‐scale prediction of intramolecular isopeptide bonds in the AlphaFold Database

We next employed a structure‐guided search method to identify putative intramolecular isopeptide bonds within predicted structures in the AlphaFold Database (AFDB; Varadi et al., 2024) using the isopeptide‐scanning software Isopeptor, which identifies isopeptide bonds in structural models using a template‐based matching approach and assigns probability scores to each hit (Costa et al., 2025). We initially tested Isopeptor's ability to detect intramolecular isopeptide bonds in AlphaFold2 (AF2) models of PDB‐deposited intramolecular isopeptide bond structures to confirm that AF2 reliably approximates the positions of isopeptide bond residues. Isopeptor identified intramolecular isopeptide bonds in 94% of AF2 models using a probability threshold of 0.65 (comparable to the recall in PDB structures; Costa et al., 2025), confirming that AF2 places intramolecular isopeptide bond residues in positions consistent with those found in PDB depositions (Figure S2).

**FIGURE 3:**
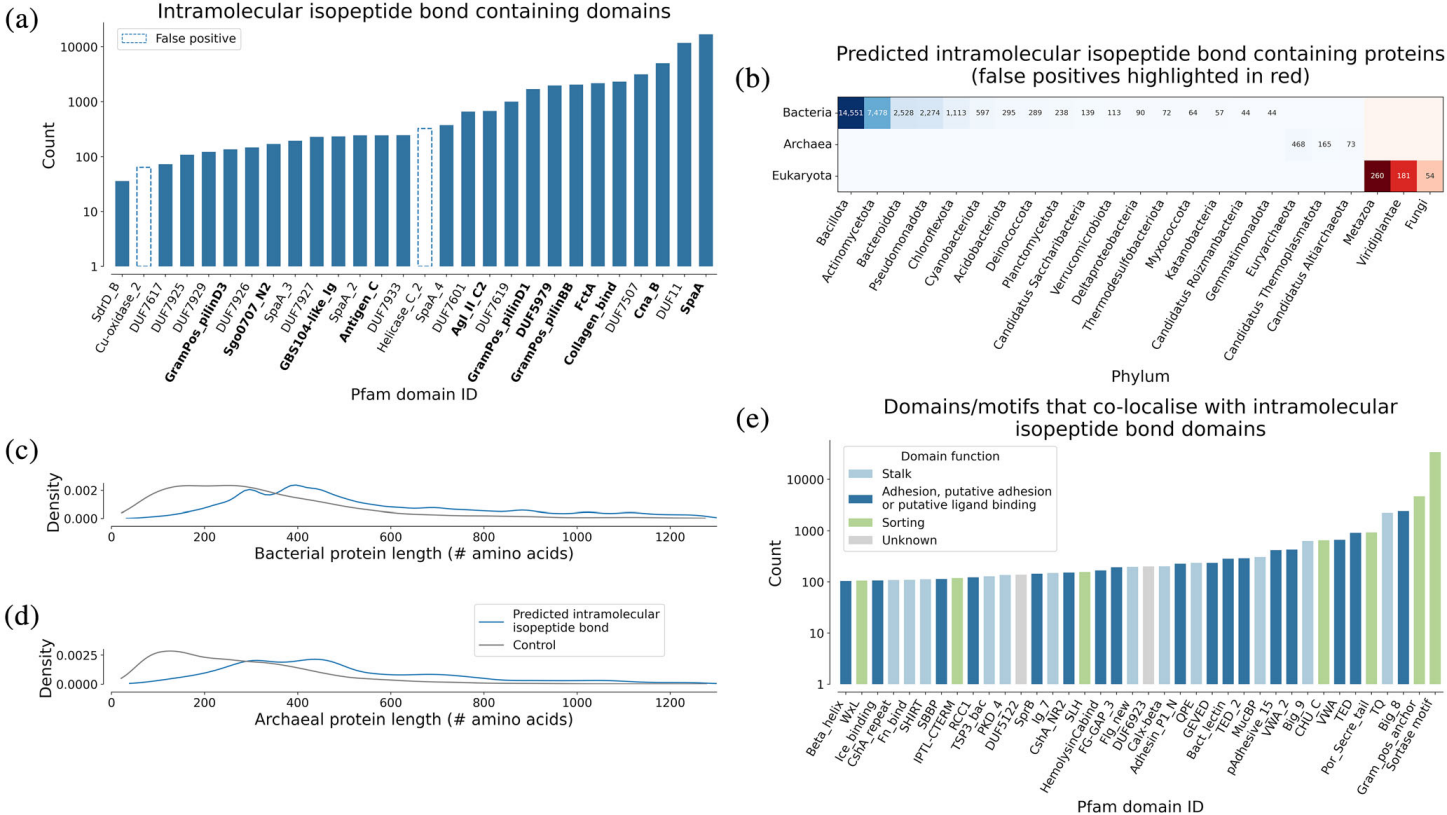
(a) Pfam domains predicted by Isopeptor to contain intramolecular isopeptide bonds in the AFDB (probability >0.65). Domains in bold were structurally characterized with an intramolecular isopeptide bond prior to this work. (b) Life domain and phylum distribution of intramolecular isopeptide bond‐containing proteins detected with Isopeptor in the AFDB (probability >0.65, phyla with <40 sequences are not shown). Eukaryotic proteins are likely false positives or are from incorrectly annotated organisms. (c, d) Sequence length distribution of bacterial and archaeal intramolecular isopeptide bond proteins detected by Isopeptor in the AFDB. Length distributions are compared with a set of 10,000 randomly selected AFDB proteins from each life domain. Note that the AFDB has an upper sequence length limit of 1280 amino acids. (e) Pfam domains co‐occurring with intramolecular IPDs identified by Isopeptor in AFDB proteins. Families were counted once per protein. Domains detected <100 times are not shown (full data are available in Figure S6), nor are domains from polypeptides annotated as eukaryotic.

Applying Isopeptor to the AFDB using a probability threshold of 0.65, we identified 69,718 intramolecular isopeptide bonds within 33,049 (0.015%) of the 214,683,839 models, all of which were located within β‐sandwich folds. Since the AFDB does not contain predictions of viral proteins, a scan was also performed against the Big Fantastic Virus Database (BFVD) to determine whether intramolecular isopeptide bonds could be detected in viral proteins (Kim et al., 2025). No hits were returned from the BFVD (data not shown). To characterize the identified intramolecular IPDs into distinct families, we mapped the AFDB Isopeptor hits to Pfam protein domains. The identified domains fell into 26 Pfam families, 12 of which had been structurally characterized with an intramolecular isopeptide bond (Figures 3a and S3, Tables 1 and S2). Multiple hits matched four pre‐existing families, two that had been annotated as probable intramolecular IPDs (DUF11 and SpaA_3), and two that had not (SdrD_B and SpaA_2). Many hits did not belong to existing Pfam families and were, therefore, used as seeds to create 10 new Pfam families. All 26 families can be grouped into three superfamilies according to the Pfam classification: the Adhesin (CL0204), Transthyretin (CL0287), and E‐set (CL0159) clans.

**TABLE 1:** Pfam domains confirmed by structural characterization (bold) or predicted (plain) to harbor intramolecular isopeptide bonds.

Pfam ID (Pfam accession code)	Pfam clan ID (accession code)‐isopeptide bond class type	PDB/AFDB accession code	PDB chain	Pfam domain start‐end	Structural domain start‐end	Experimental/predicted intramolecular isopeptide bond residues (first bond residue, catalytic residue, second bond residue)
AgI_II_C2 (PF17998)	Adhesin (CL0204) CnaA‐like	5DZ9	A	554–723	554–723	Lys 556, Asp 606, Asn 703
Sgo0707_N2 (PF20623)		4HSS	A	178–324	184–324	Lys 187, Asp 224, Asn 299
GramPos_pilinBB (PF16569)		2X9Z	A	216–328	187–325, 436–446	Lys 193, Asp 241, Asn 318
Collagen_bind (PF05737)		7LGR	A	173–306	173–317	Lys 177, Asp 210, Asn 291
Antigen_C (PF16364)		3OPU	A	1334–1492	1330–1489	Lys 1334, Asp 1383, Asn 1469
DUF7926 (PF25548)		A0A179B332	‐	188–359	190–349	Lys 193, Asp 241, Asn 334
DUF7929 (PF25551)		A0A090G7K7	‐	81–255	74–257	Lys 86, Asp 129, Asn 213
DUF7925 (PF25546)		Q8YS00	‐	259–442	260–508	Lys 264, Asp 326, Asn 415
DUF5979 (PF19407)	Transthyretin (CL0287) CnaB‐like	4BUG	A	606–718	603–718	Lys 610, Glu 680, Asn 715
SpaA (PF17802)		2XID	A	291–377	291–384, 588–598	Lys 297, Glu 347, Asp 595
GramPos_pilinD3 (PF16570)		2X9Z	A	388–448	344–433	Lys 349, Glu 405, Asn 428
FctA (PF12892)		7W7I	A	48–216	37–218	Lys 52, Glu 139, Asn 213
GramPos_pilinD1 (PF16555)		7WOI	A	48–196	48–196	Lys 57, Glu 158, Asn 195
Cna_B (PF05738)		1D2O	A	627–716	625–721	Lys 633, Glu 694, Asn 717
SpaA_2 (PF19403)		A0A086ZQG5	‐	489–625	489–629	Lys 497, Glu 571, Asn 627
SpaA_3 (PF20674)		A0A514BTT6	‐	293–411	291–412	Lys 298, Glu 353, Asn 409
SpaA_4 (PF24514)		A0A4R4IAB0	‐	700–816	696–817	Lys 704, Glu 766, Asn 814
SdrD_B (PF17210)		A0A7C3F8X4	‐	157–243	153–250	Lys 159, Glu 218, Asn 248
DUF7601 (PF24547)		B8QYD3	‐	184–307	184–308	Lys 190, Glu 263, Asn 305
GBS104‐like_Ig (PF21426)	E‐set (CL0159) CnaA‐like	3TXA	A	587–717	137–211, 587–717	Lys 188, Asp 597, Asn 692
DUF11 (PF01345)		9IFR (from this work)	A	712–812	708–828	Lys 715, Asp 748, Asn 806
DUF7507 (PF24346)		A0A2I2KYV6	‐	65–169	65–176	Lys 72, Asp 112, Asn 152
DUF7619 (PF24595)		L7WFS8	‐	653–784	655–785	Lys 657, Asp 695, Asn 764
DUF7927 (PF25549)		A0A6G8FG97	‐	120–250	119–242	Lys 125, Asp 161, Asn 225
DUF7617 (PF24593)		A0A495X870	‐	676–804	678–807	Lys 684, Asp 740, Asn 778
DUF7933 (PF25564)		A0A023BX92	‐	968–1093	968–1096	Lys 973, Asp 1008, Asn 1068

With an updated collection of intramolecular IPD families, we then surveyed their phyletic distribution in the AFDB. This revealed that intramolecular IPDs are predominantly found in bacterial entries, but we also identified hits within archaea and eukaryotes (Figure 3b). While most intramolecular isopeptide domains are found within the Gram‐positive Bacillota and Actinomycetota phyla, a significant number are found within the Gram‐negative Bacteroidota and Pseudomonadota phyla. Two previously uncharacterized DUFs were identified in Gram‐positive bacteria, Gram‐negative bacteria, and archaea: DUF11 and DUF7507, with DUF11 being by far the most broadly distributed (Figure S4). While hits were identified in eukaryotes, manual inspection revealed them to be likely false positives as the residues were solvent‐exposed, not conserved across families, and lacked homologous proteins in related eukaryotes, suggesting that these sequences may have originated from microbial contamination during sequencing projects.

We noted that the proteins identified by Isopeptor are typically longer than other proteins and often harbor tandemly repeating intramolecular isopeptide domains (Figures 3c,d and S5). These domains often co‐occur with adhesive domains, particularly Big_8 (a putative adhesion domain related to the collagen‐binding domain of the adhesin CNA; Zong et al., 2005), and also stalk domains such as the threonine‐glutamine domain (TQ), which possesses a stabilizing intramolecular ester bond (Kwon et al., 2014). Other common co‐occurring domains include Gram‐negative sorting/anchoring domains such as Por_secre_tail and CHU_C, and the Gram‐positive LPXTG cell wall anchoring motif (Figures 3e and S6). Taken together, these data suggest that a sizable portion of these intramolecular isopeptide bonds occur in elongated surface‐anchored monomeric proteins that enable adhesion, also known as fibrillar adhesins. Subsequent analysis performed using software for fibrillar adhesin detection (Monzon & Bateman, 2022) revealed that 14% of intramolecular isopeptide bond‐containing proteins identified in the AFDB by Isopeptor are likely to be fibrillar adhesins. Indeed, inspection of the Isopeptor hits identified several putative fibrillar adhesins in Gram‐positive bacteria, Gram‐negative bacteria, and archaea, covering pathogens, opportunistic pathogens, and commensal microbes (Figure S7, Table S3).

Fibrillar adhesins typically present as domain‐shuffled polypeptides with unique functionalities, dictated by the specific domain arrangements within the protein structure (Barringer et al., 2023; Monzon et al., 2021). Thus, we next characterized domains that co‐occur with intramolecular IPDs in AFDB polypeptides. Our analyses found a diverse set of co‐occurring domains which can be broadly divided into three functional categories: adhesion, stalk‐forming, and sorting domains (Figure 3e), mainly from bacteria (Figure S6). While intramolecular IPDs (primarily DUF11) were identified in archaeal AFDB proteins, co‐occurring domains were rarely detected, indicating that either these structures contain only DUF11 repeats or that co‐occurring domains do not resemble the current collection of known Pfam domain families. When other domains are present in archaea, they commonly include the Chlam_PMP domain (Pfam ID PF02415), which is also found in Chlamydia surface proteins, and PKD_4 domains (Pfam ID PF18911), which have also been detected in bacterial surface proteins. We noted that proteins of the archaeal kingdom Methanobacteriati frequently employ C‐terminal protein sorting motifs (e.g., Pfam IDs PF18204, PF26597, and PF26596), which enable covalent attachment of the C‐terminus to the cell surface by archaeosortases A (ArtA), B (ArtB), and C (ArtC), likely via attachment of lipid moieties (Haft et al., 2012).

### 2.3 DUF11 is an intramolecular IPD that is widely distributed in fibrillar adhesins of bacteria and archaea

**FIGURE 4:**
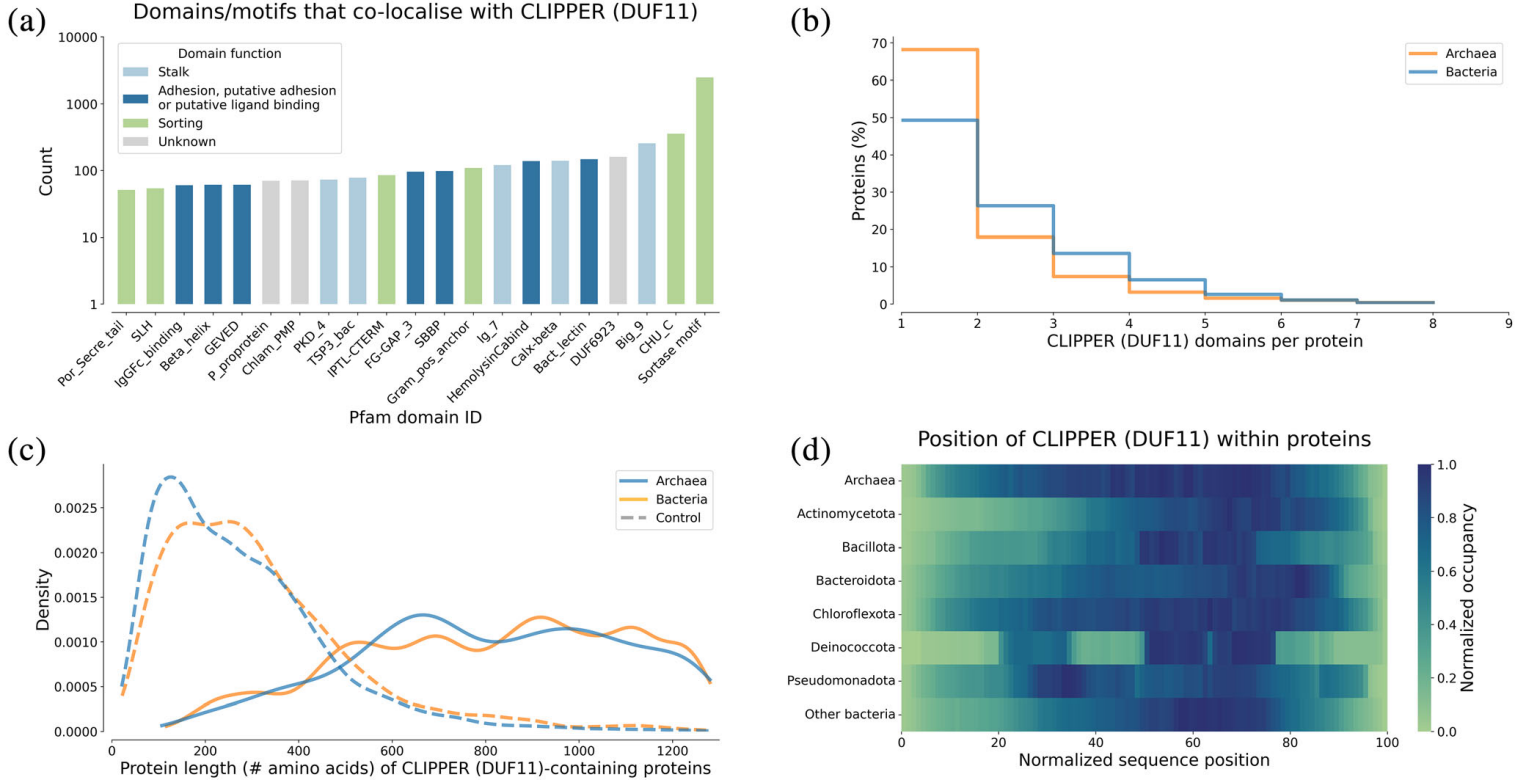
(a) Pfam domains co‐occurring with CLIPPER IPDs in bacterial and archaeal AFDB proteins (domains detected <50 times are not shown). (b) Histogram showing the number of CLIPPER IPD repeats per AFDB protein. (c) Protein length distribution of AFDB proteins with at least one CLIPPER IPD, compared to control sequences as described in Figure 3. (d) Positioning of CLIPPER IPDs within AFDB polypeptide sequences of archaea and various bacterial phyla.

The DUF11 domain family was initially deposited in the Pfam database in 1998 (Pfam ID: PF01345) and was rebuilt in 2018 to better represent several immunoglobulin‐like domains, some of which were predicted to engage in intramolecular isopeptide bond formation. AF2 predicts that DUF11 domains fold into an Ig‐like β‐sandwich structure containing a Greek‐key motif and consisting of seven to nine β‐strands. While DUF11 belongs to the E‐set Ig‐like fold clan (CL0159), it shares some topological similarity to domains of the bacterial adhesin clan (CL0204), which both represent CnaA‐like domains (Table 1). Typically, DUF11 domains are found in one or multiple copies, often tandemly repeated in the middle of long proteins harboring a signal peptide, adhesion domains, and sorting domains, indicating that the domain routinely forms the stalk of fibrillar adhesins (Figure 4). While all DUF11 domains share global sequence similarity and are predicted to exhibit a CnaA‐like fold, some DUF11 domains appear to lack intramolecular isopeptide bonds (Table S2). This heterogeneity is evident from the Pfam SEED and FULL alignments, in which the positions of the residues predicted to form isopeptide bonds are not universally conservked.

While most DUF11 domains are found in proteins from Gram‐positive and Gram‐negative bacteria, some have been identified in multiple cell‐surface proteins from archaea. Tandem DUF11 repeats are present in proteins that resemble fibrillar adhesins of the archaean genus *Methanothermobacter* (Sumikawa et al., 2019) and are present in porins (mostly without isopeptide bond signatures) that are proposed to form part of the archaeal S‐layer structure (Doloman et al., 2024). DUF11‐containing *Methanothermobacter* proteins are known to stabilize cell aggregates, and while the role of DUF11 domains in these cell proteins remains cryptic, they are suspected to be important for stabilization of the protein (Sumikawa et al., 2019). It has also been demonstrated that adhesin‐like proteins of *Methanothermobacter* species are frequently acquired as a result of lateral gene transfer from bacteria, some of which contain DUF11 domains (e.g., Msm_1533; Lurie‐Weinberger et al., 2012).

DUF11 is closely related to DUF7507, a new Pfam family built from IPDs identified by Isopeptor. DUF7507 domains are more compact than DUF11, typically consisting of seven β‐strands (as judged by the AF2 predictions). Both DUF11 and DUF7507 domains are frequently found in tandem within surface polypeptides of bacteria and archaea, often alternating between the two families in tandem. Given the likely propensity of DUF11 and DUF7507 domains to harbor intramolecular isopeptide bonds, we have renamed and refer to them hereafter as CLIPPER and CLIPPER_2 domains (Cross‐Linked IsoPeptide Protein in the Extracellular Region). Despite their wide phyletic distribution in a variety of putative host‐binding fibrillar adhesins, neither CLIPPER nor CLIPPER_2 has previously been structurally or biophysically characterized. For this reason, we proceeded to determine the structure of a member of the CLIPPER family using x‐ray crystallography to confirm the presence of an intramolecular isopeptide bond and undertook biophysical studies to characterize the thermal and proteolytic resilience of this domain.

### 2.4 Structural and biophysical characterization of a CLIPPER domain

**FIGURE 5:**
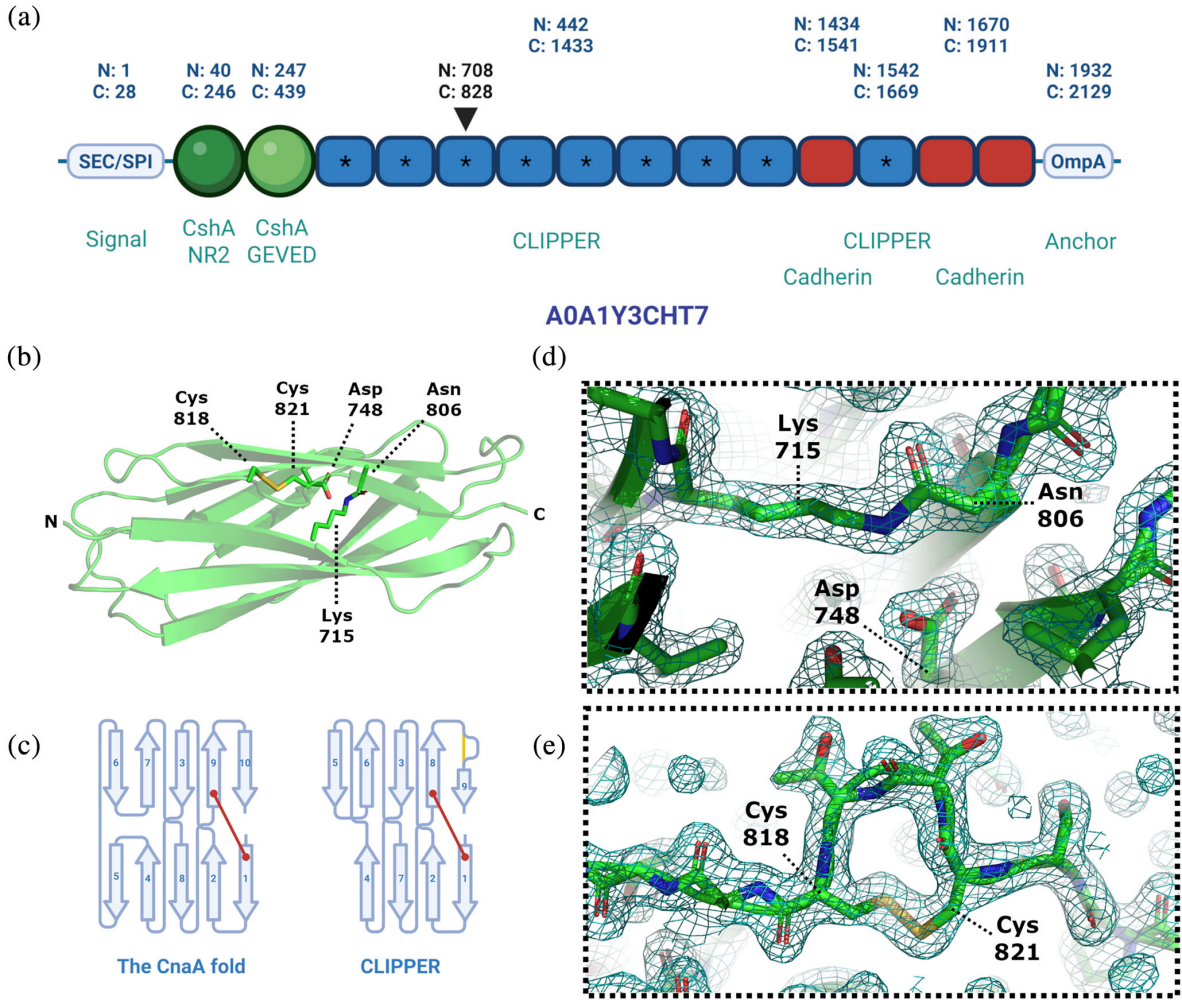
(a) Schematic of fibrillar adhesin B9T28_05395 from *Acinetobacter silvestris* ANC 4999, with N‐ and C‐termini of domain regions indicated. The black arrow indicates the CLIPPER domain investigated in this work (residues 708–828). Asterisks indicate CLIPPER domains predicted by Isopeptor to contain intramolecular isopeptide bonds. (b) A PyMOL rendering of the x‐ray crystal structure of CLIPPER_WT_, depicted in cartoon format. Residues involved in intramolecular isopeptide bond and the tetrapeptide disulfide motif formation are shown in stick format, and labeled. (c) Comparative topology diagrams of CLIPPER_WT_ and the classic CnaA fold. Isopeptide bonds are indicated by a red line, and disulfide bonds as a yellow line. (d) A zoomed‐in view of the isopeptide bond and putative catalytic aspartate of the CLIPPER_WT_ domain, depicted in stick format. (e) A zoomed‐in view of the CTTC disulfide motif. Electron density maps are shown in teal and contoured to 1σ (0.314 e/Å^3^).

We chose to structurally and biophysically characterize a CLIPPER domain from a putative fibrillar adhesin utilized by a Gram‐negative bacterium within a well‐characterized, high‐quality genome. To this end, we identified a putative fibrillar adhesin 2129 amino acids in length from the genome of *Acinetobacter silvestris* ANC 4999 (Nemec et al., 2022), named B9T28_05395 (Uniprot ID: A0A1Y3CHT7_9GAMM). This fibrillar adhesin appears to be composed of an N‐terminal adhesive CshA_NR2‐like domain, a CshA_GEVED‐like domain, a tandem repeat stalk of either CLIPPER domains or C‐terminal cadherin‐like domains, and an OmpA‐like anchoring domain (Figure 5a). The CLIPPER repeat between residues 708–828 demonstrated the highest average identity to the other CLIPPER repeats (data not shown) and was subsequently chosen for experimental characterization.

Constructs of wild‐type CLIPPER (CLIPPER_WT_) and a variant lacking the predicted isopeptide‐forming lysine (CLIPPER_K715A_) were expressed, purified (Figure S8), and their His‐tag cleaved off prior to crystallization attempts. Following sparse‐matrix screening of various crystallization conditions, CLIPPER_WT_ crystals were acquired, with data extending to 1.77 Å resolution (Table 2). The final modeled structure consists of a β‐sandwich formed by two antiparallel sheets of four and five β‐strands (Figure 5b). The domain presents as a CnaA‐like fold (Figure 5c) and exhibits an intramolecular isopeptide bond between Lys‐715 and Asn‐806, presumably catalyzed by nearby Asp‐748 (Figure 5d). The final β‐strand (β9) of the fold exhibits a mid‐strand β‐bulge that is caused by a tetrapeptide disulfide motif of Cys‐818/Thr‐819/Thr‐820/Cys‐821 (Figure 5e). The observation of a tetrapeptide disulfide bond motif prompted us to investigate the wider disulfide prevalence in intramolecular IPDs. We found that disulfide bonds co‐occur alongside isopeptide bonds in 29.7% and 34.9% of CLIPPER and CLIPPER_2 domains respectively at various sites. We also noted that disulfide bonds are prevalent in IPD families SpaA_2, SpaA_3, SpaA_4, and DUF7933 domains (Table S4). They are predominantly found in the Gram‐positive Actinomycetota (in 41.3% of intramolecular IPDs), and in Gram‐negative Acidobacteriota (79.2%), Pseudomonadota (58.2%), and Chloroflexota (42.7%; Table S5).

**TABLE 2:** Crystallography data collection and refinement statistics for the CLIPPER domain (residues 708–828 of B9T28_05395).

Crystallography data collection and refinement	
Data collection	
Space group	*P6*_*3*_
Unit cell dimensions (Å): A, B, C	93.859, 93.859, 21.994
Unit cell angles (°): *α*, *β*, *γ*	90, 90, 120
Datasets merged	2
Resolution (Å)	81.28–1.77 (1.80–1.77)
CC1/2 (%)	0.993 (0.401)
R_pim_	0.161 (3.626)
Number of unique reflections	11,209 (547)
Multiplicity	38.7 (29.5)
Overall signal‐to‐noise ratio (I/*σ*)	5.1 (0.4)
Completeness (%)	99.91 (97.16)
Refinement	
*R*work	0.200
*R*free	0.209
No protein atoms used in refinement	876
No water atoms used in refinement	113
*B* factors for protein atoms (Å_2_)	31.89
*B* factors for water atoms (Å_2_)	36.29
RMS deviations—length (Å)	0.015
RMS deviations—angle (°)	1.544
Ramachandran‐favored residues (%)	97
Ramachandran outlying residues (%)	1

*Note*: Values in parentheses are for the highest resolution shell (CC_1/2_ > 0.4 used to decide resolution cut‐off).

**FIGURE 6:**
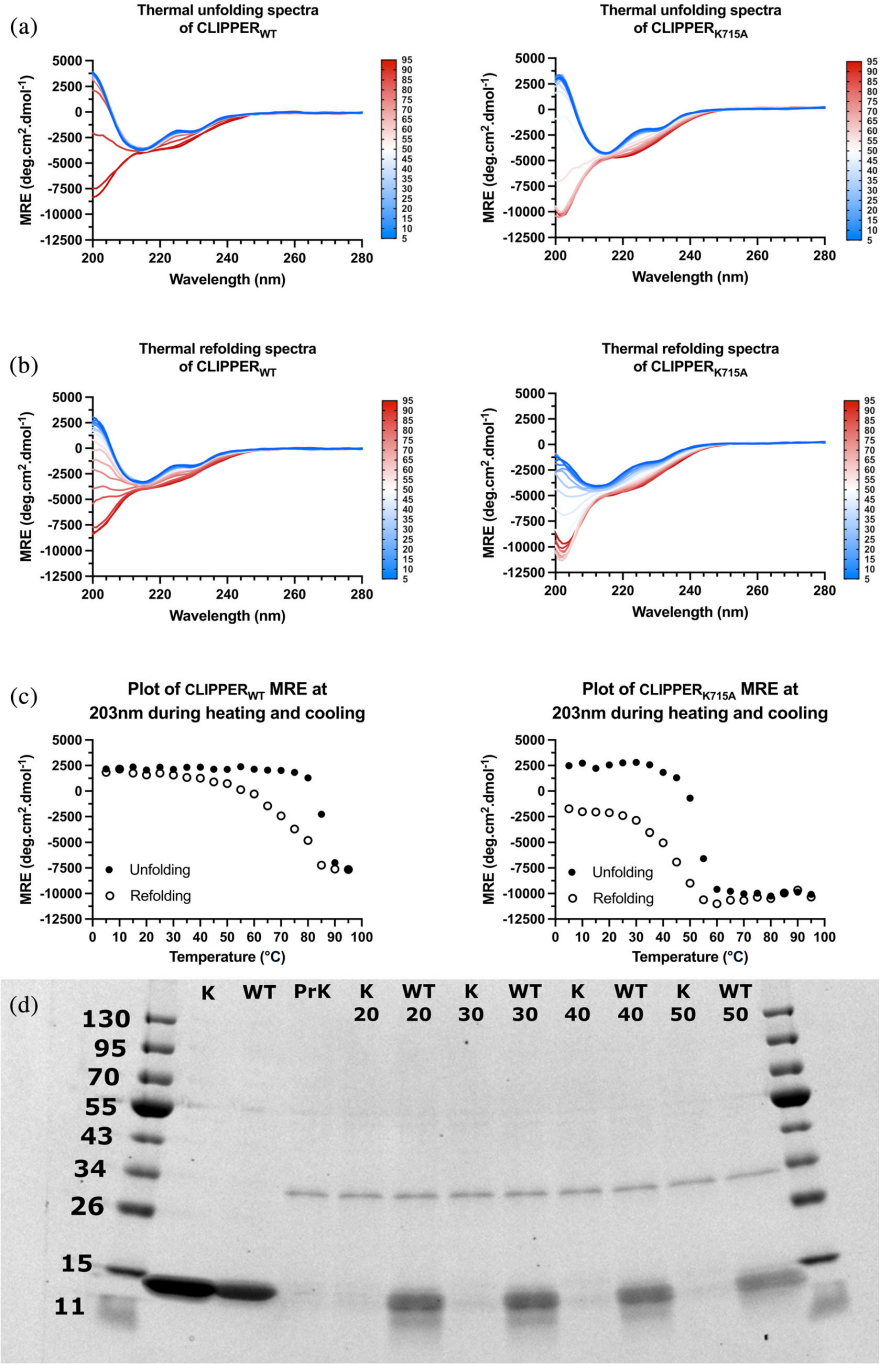
(a) CD thermal unfolding spectra of CLIPPER_WT_ and CLIPPER_K715A_, increasing from 5°C to 95°C with spectra recorded at 5°C increments. (b) CD thermal refolding spectra of CLIPPER_WT_ and CLIPPER_K715A_, decreasing from 95°C to 5°C with spectra recorded at 5°C increments. (c) 203 nm MRE plot of CLIPPER_WT_ and CLIPPER_K715A_ during thermal unfolding and refolding. (d) SDS‐PAGE analysis of CLIPPER_WT_ (WT) and CLIPPER_K715A_ (K) polypeptides after incubation with buffer or Proteinase K (PrK) at the indicated temperatures (in degrees Celsius) for 1 h.

We next sought to characterize the stabilizing effects of the intramolecular isopeptide bond on CLIPPER. Since intramolecular isopeptide bonds usually impart significant thermotolerance to CnaA‐like domains (Heidler et al., 2021), we investigated the thermal stability of CLIPPER_WT_ and the isopeptide‐lacking CLIPPER_K715A_ using circular dichroism (CD) by collecting spectra from 5°C to 95°C at 5°C intervals (Figure 6a). CLIPPER_WT_ and CLIPPER_K715A_ demonstrate comparable β‐sheet‐like spectra, with a negative mean residue ellipticity (MRE) at 218 nm and positive MRE at <200 nm, indicating that both constructs are folded. At higher temperatures, a transition to a disordered state is observed for both constructs, at ~80°C for CLIPPER_WT_ and ~45°C for CLIPPER_K715A_ (Figure 6c). At high temperatures, the MRE values at 203 nm appear to differ between the polypeptides, reaching a plateau of ~−7500 deg.cm^2^.dmol^−1^ in CLIPPER_WT_ and ~−10,000 deg cm^2^ dmol^−1^ in CLIPPER_K715A_ (Figure 6a,c), indicating that the intramolecular isopeptide bond may prevent total unfolding of the polypeptide chain.

We then assessed whether the intramolecular isopeptide bond facilitates refolding from thermally denatured states. To this end, the samples were subsequently cooled from 95°C to 5°C, with spectra collected at 5°C intervals (Figure 6b,c). During cooling, CLIPPER_WT_ appears to regain a predominantly β‐sheet structure, whereas CLIPPER_K715A_ does not, with MRE between 200 and 205 nm failing to achieve a positive value. When plotting the folded‐unfolded‐refolded transition at 203 nm, both constructs demonstrate rapid unfolding but gradual refolding (which is incomplete for CLIPPER_K715A_; Figure 6c). These data indicate that the intramolecular isopeptide bond of CLIPPER bestows significant thermotolerance to the domain and enables efficient refolding of the domain upon cooling.

Finally, we sought to test the proteolytic susceptibility of CLIPPER_WT_ and CLIPPER_K715A_ to determine whether the intramolecular isopeptide bond imparts proteolytic resistance (as observed in other intramolecular IPDs; Kang & Baker, 2009). Recombinant CLIPPER_WT_ and CLIPPER_K715A_ polypeptides were incubated with Proteinase K at 20°C, 30°C, 40°C, and 50°C for 1 h and visualized using sodium dodecyl sulfate polyacrylamide gel electrophoresis (SDS‐PAGE). The results revealed that CLIPPER_WT_ is significantly more resistant than CLIPPER_K715A_ to proteolysis when treated with Proteinase K over all temperatures (Figure 6d), indicating that the presence of the isopeptide bond confers significant proteolytic resilience to the fold, in line with the properties of other intramolecular IPDs (Kang & Baker, 2009).

## 3 DISCUSSION

To resist the significant mechanical forces at play during adherence to a surface (Busscher & van der Mei, 2006), microbial adhesins are known to employ various strategies to stabilize the extended protein and resist various stresses. While some strategies employ non‐covalent interactions for this purpose (Lipke, 2025; Vance et al., 2020), covalent cross‐links have also been postulated to provide significant resilience to adhesins under mechanical stress. Such covalent strategies include intramolecular isopeptide bonds (Alegre‐Cebollada et al., 2010; Echelman et al., 2016), intramolecular ester bonds (Lei et al., 2021), and intermolecular thioester bonds, which covalently bind host surfaces and maintain adherence under strong mechanical forces (Alonso‐Caballero et al., 2020; Walden et al., 2015).

This work characterized common environmental features surrounding intramolecular isopeptide bonds and surveyed their distribution in nature. Our analyses quantitatively confirm previous observations that these bonds are invariably found within hydrophobic cores, often near aromatic residues (Figure 2; Kang et al., 2007; Kang & Baker, 2009). We found that many intramolecular isopeptide bonds position the lysine Nζ atom in a pose resembling “above ring” amino‐aromatic interactions (stacked interactions within 5 Å of the ring centroid and within 50° of the normal of the aromatic plane, Singh & Thornton, 1990; Mitchell et al., 1994). These “aromatic caps” (as referred to herein) are detected in 84% and 35% of CnaA‐like and CnaB‐like domains respectively, in 94% and 24% of isopeptide bonds in *cis*/*trans* conformations, and are notably absent in folds containing Lys‐Asp bonds. Their prevalence may suggest that aromatic caps play a functional role in intramolecular IPDs. For example, aromatic caps might function as a recruitment site for isopeptide‐forming side chains, creating positions conducive to bond formation during protein folding. Alternatively, the aromatic planes may interact with polar moieties of the isopeptide bond residues in stacked orientations to reduce entropy within the hydrophobic core. Future work investigating the rate of isopeptide bond formation and domain entropy in aromatic vs. non‐aromatic folds may clarify whether they play a functional role in isopeptide bond formation and domain stabilization.

In more than half of the surveyed structures, we found electron densities consistent with water or ammonia molecules proximal to isopeptide Asn_Oδ_/Asp_Oδ_ atoms. It is not clear whether the presence or absence of such molecules reflects distinct optimizations of the chemical environment. It may be that their frequent occurrence within the domain core represents channels that enable the release of byproducts upon bond formation (as suggested previously, Hagan et al., 2010; Hu et al., 2011). Alternatively, they may play a stabilizing role within the domain interior via key hydrogen‐bond interactions.

Intramolecular IPDs appear to group closely within three superfamily clans. Two extant domain families had not been annotated as intramolecular IPD families prior to this work (SdrD‐B and SpaA_3), while two (DUF11 and SpaA_3) were annotated as such in the Pfam database but lacked experimental validation. Ten new families were created, expanding the repertoire of known intramolecular isopeptide domain families to 26 (Table 1). Considering that all 26 families are predicted to resemble β‐sandwich folds with Greek‐key motifs, it is likely that CnaA‐like and CnaB‐like folds share a common evolutionary origin. While our results have not identified intramolecular isopeptide bonds in non‐β‐sandwich folds, recent work has introduced an autocatalytic intramolecular isopeptide bond to a β‐sandwich fold lacking the Greek‐key motif, indicating that such bonds can exist in non‐CnaA/CnaB‐like folds (Srisantitham et al., 2025). Indeed, other work has described a naturally occurring autocatalytic intermolecular isopeptide bond in an α‐helical hairpin fold (Remaut, Sleutel & Sogues 2025), indicating that α‐helical folds are capable of forming isopeptide bonds. Future studies employing the findings of this work and protein design tools may elucidate whether isopeptide bonds can be engineered into alternative synthetic folds to generate stabilized synthetic domains for novel adhesive and material technologies.

Until now, only one intramolecular IPD has been structurally characterized from an organism that is not a Gram‐positive bacterium (Heidler et al., 2021), and their wider phyletic distribution has remained uncertain (Schwarz‐Linek & Banfield, 2014). We found that intramolecular isopeptide bonds are prevalent in Gram‐positive bacteria, but that three families demonstrate wider distribution. DUF11 (renamed CLIPPER) and the closely related domains DUF7507 (renamed CLIPPER_2) and DUF7619 are frequently found in polypeptides of Gram‐negative bacteria, while CLIPPER and CLIPPER_2 also occur in archaeal proteins (Figure S4). We found that tandemly repeating intramolecular IPDs are usually located in elongated cell‐surface proteins of host‐binding pathogens, opportunistic pathogens, and commensal microbes (Figures 3, S7, and Table S3). Notably, CLIPPER appears to be the most widely distributed domain, often tandemly repeated in the stalks of fibrillar adhesins (Figures 4 and S4). The frequent use of tandemly repeating intramolecular isopeptide bonds in fibrillar adhesins of host‐binding bacteria and archaea indicates that they may be important for the efficient colonization of host tissues and may, therefore, present attractive targets for novel antimicrobial therapeutics. Future work may focus on generating novel therapeutics to interfere with isopeptide bond formation and abrogate host colonization by pathogens, following previous work that has demonstrated promising results employing this strategy (Rivas‐Pardo et al., 2018).

Given that CLIPPER was found to be the most broadly distributed intramolecular isopeptide domain from our work, we experimentally characterized an exemplary domain from the fibrillar adhesin B9T28_05395 of *Acinetobacter silvestris* ANC 4999. Our crystal structure reveals that the CnaA‐like fold harbors an intramolecular isopeptide bond between Lys‐715 and Asn‐806, presumably catalyzed by Asp‐748 (Figure 5). The isopeptide bond enables significant thermostability and resistance to proteolysis and can refold after thermal denaturation, properties that were abrogated in an isopeptide‐lacking variant (Figure 6). This indicates that the isopeptide bond of CLIPPER domains enables significant resilience to stress, which is likely of functional importance when present in the stalks of fibrillar adhesins from Gram‐positive bacteria, Gram‐negative bacteria, and archaea.

This body of work indicates that intramolecular isopeptide bonds are broadly utilized in nature to stabilize adhesin stalks faced with various stresses. Whether other covalent intramolecular bonds are as widely distributed is not currently known. Further work investigating the distribution of such bonds in the AFDB may probe their phyletic distribution in nature, reveal key features of their chemical environments, and inform design processes aiming to introduce covalent intramolecular bonds into synthetic folds.

## 4 MATERIALS AND METHODS

### 4.1 Analysis of intramolecular isopeptide bond structural features

Wild‐type PDB structures containing an intramolecular isopeptide bond, with x‐ray diffraction resolution ≤2.5 Å and no unusual residue properties (e.g., incomplete or missing isopeptide bond triad side chains present on flexible loops), were chosen from the previously collated collection of intramolecular IPDs (Costa et al., 2025). This dataset includes remodeled structures of intramolecular isopeptide bonds, which have been deposited in the PDB with incorrect isopeptide bond geometries. Only one PDB entry was assessed per sequence‐identical domain, considering the 20 residues flanking each side of the first and last isopeptide bond signature positions, unless specified otherwise. rASA was calculated using the Biotite package v1.3.0 (Kunzmann & Hamacher, 2018) sasa function with point_number 500 and values normalized using Rost and Sander maximum ASA values (Rost & Sander, 1994). Bonds were classified as *cis* for pseudo ω angles <60° or *trans* for angles >120°. “Aromatic caps” were identified as aromatic residues with the centroid of their aromatic ring within 5 Å of the isopeptide bond Lys_Nζ_ atom, where the angle between the centroid‐Lys_Nζ_ atom vector was <50° or >130° from the normal of the aromatic ring plane (in the case of tryptophan, the closest of the two rings was considered). Where no aromatic caps were detected, the closest aromatic within 10 Å of the Lys_Nζ_ atom was plotted. The analyses were conducted with custom python v3.12.2 scripts with the Biotite, Pandas v2.1.1 (McKinney, 2010), seaborn v0.13.2 (Waskom, 2021), matplotlib v3.8.0 (Hunter, 2007), biopython v1.83 (Cock et al., 2009), and Numpy v1.26.4 (Harris et al., 2020) packages for calculations.

### 4.2 AlphaFold2 modeling

AlphaFold2 modeling was performed on A100 GPUs with AlphaFold version 2.3.1 (Jumper et al., 2021) (https://github.com/kalininalab/alphafold_non_docker) and cuda version 11, with active amber relaxation and PDB templates options. Multiple sequence alignment (MSA) was performed on the sequence database suggested from the GitHub webpage on July 2021 (https://github.com/kalininalab/alphafold_non_docker). Missing residues from PDB sequences were replaced with glycine for AF2 predictions. Predictions generated 5 models, and the highest pLDDT‐scoring model was selected.

### 4.3 Large‐scale prediction of intramolecular isopeptide bonds using Isopeptor, Pfam mapping and creation of new Pfam domains

Isopeptor version v0.0.75 was installed using the pip python package manager and run with default parameters, employing a probability threshold of >0.65 against the AFDB version 4 and the BFVD version 2023_02. AFDB domains detected by Isopeptor were mapped to Pfam version 37_0 entries and assigned to Pfam domains when entries covered at least two of the three residues required for intramolecular isopeptide bond formation. Sortase motifs were annotated within isopeptide bond‐containing proteins using the following regex expressions within the last 50 C‐terminus amino acids: LP.T[G|A|N|D], NP.TG, LP.GA, LA.TG, NPQTN, IP.TG. Newly created Pfam domains were mapped to AFDB proteins using hmmscan (HMMER version 3.3.2, Eddy, 2011) and filtered considering annotated domain and sequence gathering thresholds. In cases of conflicting annotations, priority was given to domains annotated in Pfam version 37_0. AFDB hits with no corresponding Pfam annotation were used to create new Pfam domain families via sequence clustering, using N‐terminal domain boundaries −10/−5 residues from the isopeptide‐bonded lysine, and C‐terminal domain boundaries +5/+30 residues from the isopeptide‐bonded asparagine/aspartate for CnaB‐like/CnaA‐like domains, respectively. Clustering was performed using MMseqs2 software version 17.b804f (Steinegger & Söding, 2017) via the easy‐cluster command and the following flags: cluster‐mode 2, cov‐mode 0, min‐seq‐id 0.25, coverage 0.9, and aligned using MAFFT or Muscle (Edgar, 2004; Katoh & Standley, 2013). The resultant MSAs were used as initial seeds to search the reference proteome database using hmmsearch (HMMER version 3.3.2). Pfam families were built via repeated iterative searches and manual refinement of boundaries, member selection, and inclusion thresholds (which were adjusted for each family to exclude false positives and optimize signal‐to‐noise).

### 4.4 Proximity analysis

For the proximity analysis, we considered amino acid side chains within 6 Å of the centroid formed by the α‐carbons of each isopeptide bond triad. β‐carbons were excluded from all amino acids except alanine in order to avoid capturing distant side chains that point towards the solvent. The analysis was conducted on the non‐redundant set of PDB structures described previously using a custom Python script with Biotite, Biopython, Numpy, and Pandas packages for calculations (see above for versions).

### 4.5 Assessing fibrillar adhesin prevalence

For the prediction of fibrillar adhesin prevalence, the FAL_prediction software was downloaded and employed (https://github.com/VivianMonzon/FAL_prediction). FAL_prediction was employed against full‐length sequences of Isopeptor‐identified AFDB proteins, using a probability threshold of >0.9. The Iupred2a and T‐Reks dependencies were downloaded from https://iupred2a.elte.hu/download_new (Mészáros et al., 2018) and https://bioinfo.crbm.cnrs.fr/index.php?route=tools&tool=3 (Jorda & Kajava, 2009), respectively.

### 4.6 Detection of disulfide bonds

For the detection of disulfide bonds, disulfide bonds were assigned to cysteine residues with sulfur atoms within an atomic distance lower than the sum of their van der Waals atom radii (i.e., clashing; Bondi, 1964). The analysis was conducted on structures of putative intramolecular isopeptide bond‐containing proteins obtained from the AFDB by Isopeptor using a custom python script with the biopython and Pandas packages for calculations (see above for versions).

### 4.7 CLIPPER expression

A synthetic gene encoding residues 708–828 of *Actinetobacter silvestris* ANC4999 fibrillar adhesin B9T28_05395 (Uniprot accession A0A1Y3CHT7, “CLIPPER_WT_”) and a mutant lacking the isopeptide‐forming lysine residue (“CLIPPER_K715A_”) were ordered from Thermo Fisher Scientific (Table S6), codon‐optimized for *E. coli*. Synthetic genes were inserted into HindIII/KpnI‐linearized pOPINF expression vector following polymerase chain reaction with appropriate primers, using the In‐Fusion™ kit (Takara Bio), following the instructions of Berrow et al., 2007 and verified via DNA sequencing (Eurofins Genomics). The encoded constructs contain an N‐terminal hexa‐histidine tag with a 3C cleavage site, and were transformed into BL21(DE3) *Escherichia coli* (Thermo Fisher Scientific) using heat‐shock, grown in 1 L cultures of Luria Broth supplemented with carbenicillin at 100 μg mL^−1^ until an optical density of 0.8, and induced via addition of isopropyl β‐D‐1‐thiogalactopyranoside to a concentration of 1 mM. Cultures were incubated for 16 h at 18°C (180 RPM), harvested by centrifugation (4500 × g, 30 min), the pellets flash‐frozen in liquid nitrogen, and stored at −80°C.

### 4.8 CLIPPER purification

Pellets were resuspended in phosphate‐buffered saline (PBS, 137 mM NaCl, 3 mM KCl, 10 mM Na_2_HPO_4_, 1.8 mM KH_2_PO_4_, pH 7.4), lysed using a 120 sonic dismembrator (Fisher Scientific, amplitude 70%, 20 s on, 20 s off, 10 min), and centrifuged (39,000 × g, 30 min). Supernatant was applied to a 5 mL HisTrap Nickel‐NTA column (Cytiva) equilibrated with PBS + 20 mM Imidazole, pH 7.4. A concentration gradient of 20 mM to 500 mM imidazole in PBS was applied over 60 mL for 1 h. Fractions were collected, concentrated, and subjected to size exclusion chromatography (SEC) purification using an EnRich SEC 650 10 × 300 column (BIORAD) equilibrated with PBS for circular dichroism and proteolysis assays or Tris‐buffered saline (TBS, 20 mM Tris, 100 mM NaCl, pH 8.0) for crystallization studies. SEC fractions were subjected to SDS‐PAGE analysis, mixing 10 μL with 10 μL of 2× Laemli buffer (BioRad), heated at 95°C for 5 min, applied to Novex Tris‐Glycine precast SDS‐PAGE gels (Thermo), and subjected to 200 V for 30 min in an X‐Cell SureLock system (Thermo), and visualized using Instant Blue stain (Fisher Scientific).

### 4.9 CLIPPER crystallization and data collection

His‐tags of CLIPPER_WT_/CLIPPER_K517A_ were cleaved using HRV3C protease (Takara Bio), removed via application to a HisTrap column, and repurified using SEC. Sitting‐drop vapor‐diffusion sparse matrix screens of CLIPPER_WT_/CLIPPER_K517A_ at 5–10 mg mL^−1^ were set up using commercial screens (Molecular Dimensions) and produced crystals in both SG1 screen (condition F1) and Structure Screen 1 + 2 (condition A3). Following optimization, well‐diffracting crystals were grown in 1:1 droplet ratios of 5 mg mL^−1^ CLIPPER_WT_ with 0.2 M ammonium sulfate, 0.2 M sodium/potassium tartrate, 0.1 M sodium acetate, pH 5.5 in 4 μL droplets (CLIPPER_K715A_ yielded no crystals). Crystals were mounted in LithoLoops (molecular dimensions), flash‐cooled in liquid nitrogen without cryoprotectant, and diffraction data collected at the i24 beamline of Diamond Light Source, UK. Data were processed using Xia2 and DIALS (Winter, 2010; Winter et al., 2018) and phases calculated using molecular replacement in CCP4i2 with MOLREP (Potterton et al., 2018; Vagin & Teplyakov, 1997) using the CLIPPER_WT_ AF2 model and iteratively refined via manual model building with REFMAC and COOT (Emsley et al., 2010; Vagin et al., 2004). The model and structure factors for CLIPPER_WT_ have been deposited at the PDB with the code 9IFR.

### 4.10 Circular dichroism

A volume of 1 mL aliquots of CLIPPER_WT_/CLIPPER_K517A_ polypeptides were dialyzed overnight at 4°C in 5 L of circular dichroism (CD) buffer (10 mM sodium phosphate, 100 mM sodium fluoride, pH 7.4) using SnakeSkin dialysis tubing (10 kDa cutoff, Thermo). CD spectra were recorded for CLIPPER_WT_ and CLIPPER_K517A_ using a Jasco J‐1500 spectrophotometer continuously purged with nitrogen and fitted with a Peltier temperature control unit. Spectra were obtained from sample volumes of 300 μL in a cuvette with a 1 mm path length (Hellma Analytics) with protein concentrations of 0.36 mg mL^−1^ (CLIPPER_WT_) and 0.38 mg mL^−1^ (CLIPPER_K715A_) after blanking with CD buffer from the dialysis bucket. Spectra were recorded from 5°C to 95°C at 5°C intervals (thermal unfolding) and subsequently 95–5°C (thermal refolding) over a spectral range of 180–280 nm. High‐tension threshold (HT) voltage was continuously recorded to ensure HT was <600 V. Spectra were averaged from four repeat scans and smoothed using a Savitsky–Golay smoothing algorithm over a window of 11 data points.

### 4.11 Proteolysis assay

Proteinase K from *Tritirachium album* was used for the proteolysis assay (Sigma Aldrich). A volume of 10 μL of 1 mg mL^−1^ CLIPPER_WT_/CLIPPER_K517A_ was mixed with 10 μL of 1 mg mL^−1^ Proteinase K in PBS and aliquoted into thin‐walled PCR tubes (StarLab) on ice. After mixing, aliquots were placed inside a Sensoquest LabCycler (Geneflow) along a heated gradient at 20°C, 30°C, 40°C, or 50°C for 1 h. Aliquots were immediately mixed with SDS running buffer and subjected to SDS‐PAGE analysis.

## AUTHOR CONTRIBUTIONS

**Francesco Costa:** Conceptualization; data curation; formal analysis; investigation; methodology; software; validation; visualization; writing – original draft; writing – review and editing. **Ioannis Riziotis:** Conceptualization; methodology; software; validation. **Antonina Andreeva:** Conceptualization; data curation; formal analysis; investigation; methodology; visualization; writing – original draft; writing – review and editing. **Delhi Kalwan:** Investigation; writing – review and editing. **Jennifer de Jong:** Investigation; writing – review and editing. **Philip Hinchliffe:** Formal analysis; validation; writing – review and editing. **Fabio Parmeggiani:** Funding acquisition; resources; supervision; writing – review and editing. **Paul R. Race:** Conceptualization; funding acquisition; resources; supervision; writing – review and editing. **Steven G. Burston:** Conceptualization; methodology; supervision; writing – review and editing. **Alex Bateman:** Conceptualization; funding acquisition; methodology; project administration; writing – original draft; writing – review and editing. **Rob Barringer:** Conceptualization; data curation; funding acquisition; investigation; methodology; project administration; supervision; visualization; writing – original draft; writing – review and editing.

## FUNDING INFORMATION

This work was supported by the UKRI Biotechnology and Biological Sciences Research Council (BB/X012492/1, BB/W013959/1, BB/T008741/1, and BB/T001968/1), EMBL core funds, and a UKRI Engineering and Physical Sciences Research Council Impact Accelerator Award (EP/X525674/1).

## CONFLICT OF INTEREST STATEMENT

The authors declare no conflicts of interest.

## Supporting information


**Table S1.** Prevalence of proximal waters <5 Å of intramolecular isopeptide bonds. Only one PDB entry was assessed per sequence‐identical domain.
**Table S2.** Pfam domains detected with Isopeptor and total counts of domains from the AFDB. Domain assignment was executed as explained in the results section. Only domains detected at least 20 times are shown. False positives have been excluded.
**Table S3.** A list of human‐binding pathogens/opportunistic pathogens that employ cell‐surface proteins containing intramolecular IPDs identified by Isopeptor.
**Table S4.** Number and percentage of domains predicted to contain intramolecular isopeptide and disulfide bonds.
**Table S5.** Number of domains predicted to contain both isopeptide and disulfide bonds and their % relative to the total number of IPDs for each phylum. Phyla with less than 100 counts are reported under “other.”
**Table S6.** Sequences of synthetic DNA used in this study, and the source of pOPINF plasmid used for protein expression. pOPINF‐complementary sequences of primers are underlined in bold.
**Figure S1.** (A) Average number of amino acids within 6 Å of the isopeptide bond centroids from PDB structures, per domain. Isopeptide bonds favor neighboring hydrophobic residues, especially aromatic ones: tyrosine in CnaA‐like domains and phenylalanine in CnaB‐like domains. (B) Scatterplot showing the distance from the isopeptide bond Nζ atoms to the closest aromatic ring and the angle between the aromatic ring and the isopeptide Cγ‐Nζ planes. The minimal distance to either the closest aromatic ring atom or to the aromatic ring center is reported. A minority of proximal aromatic rings (distance <3.8 Å) interact with the isopeptide Nζ atom with a conformation compatible with H‐bonding (interplanar angle around 90°; example a. PDB ID: 2X9W) while most of them engage in stacking interactions (planar angle <30° or planar angle >150°; example b. PDB ID 3GLE; Mitchell et al., 1994). Sequence redundancy was not removed in panel (B).
**Figure S2.** (A) An example in which the intramolecular isopeptide bond modeled by AlphaFold2 resembles the bond of the PDB structure (PDB ID: 5XCB). (B) An example in which the intramolecular isopeptide bond modeled by AlphaFold2 does not resemble the one found in the PDB structure (PDB ID: 6M3Y). For panels (A) and (B), both intramolecular isopeptide bonds are confidently predicted by Isopeptor (probability 0.89 and 0.73, respectively). The average pLDDT of isopeptide bond side chains in the AlphaFold2 models is >90 (see Figure S5A). (C) Isopeptide bond length distributions as calculated between the atoms that form the covalent link (Lys_Nζ_ and Asp/Asn_Cɣ_). (D) Dihedral pseudo ω angle distributions. (E) pseudo ψ and φ angle distributions. Pseudo dihedral angles were calculated as described in Costa et al., 2025. The difference in distances between asparagine/aspartate and lysine side chains in AF2 models likely reflects an attempt to prevent local clashes. This is also reflected in the distribution of pseudo ω angles (in which the *cis* conformation is unfavorable in AlphaFold2 models). Sequence redundancy removal was not applied in this figure.
**Figure S3.** Distribution of Isopeptor probabilities for domains which contain an intramolecular isopeptide bond and for domains unlikely to contain an isopeptide bond. Intramolecular IPDs exhibit a higher Isopeptor probability score (above the threshold of 0.65), indicating that Isopeptor is detecting isopeptide bonds with high recall. There are a few exceptions to this: SpaA_2, SpaA_3 and SdrD_B domains have a substantial fraction of isopeptide bonds predicted with low confidence, likely due to a lack of experimental templates closely matching their isopeptide bond geometries. Helicase_C_2 and Cu‐oxidase_2 domains, which have a substantial fraction of isopeptide bond signatures above the probability threshold of 0.65, were determined to be false positives.
**Figure S4.** Distribution of intramolecular IPDs per phylum and superkingdom, detected with Isopeptor.
**Figure S5.** (A) Distribution of pLDDT values for predicted intramolecular isopeptide bond residues across the AFDB. (B) Intramolecular isopeptide bonds detected per protein. 57% and 52% of bacterial and archaeal proteins detected by Isopeptor contain more than one isopeptide bond, respectively.
**Figure S6.** Domains co‐occurring with intramolecular IPDs, divided into functional classes and phylum. The most common functional classes are adhesion, sorting and stalk.
**Figure S7.** A selection of AFDB proteins predicted to contain intramolecular isopeptide domains, identified by Isopeptor. Adhesive domains are depicted as circles, stalk domains are depicted as rounded boxes, and anchor motifs/domains and signal peptides are depicted in light blue boxes at the C and N termini, respectively. Domains are color coded by domain identity. Dotted outlines indicate domains that could not be correlated to Pfam domain families using sequence information, but that exhibit predicted structures that are similar to other domains of known function. Domain families are listed below each domain or domain repeat region. TED = Thioester Domain, TQ = T‐Q ester bond domain, Coll bind = Collagen binding domain, CAM like = Cell adhesion module‐like domain, CBD like = Carbohydrate binding‐like domain, Lec like = Lectin‐like domain, Cadh like = Cadherin‐like domain.
**Figure S8.** (A) SEC chromatograms of CLIPPER_WT_ and CLIPPER_K715A_, demonstrating one primary peak per polypeptide indicative of a monomer. (B) SDS‐PAGE analysis of eluted SEC fractions of CLIPPER_WT_ (left) and CLIPPER_K715A_ (right).

## Data Availability

The data that support the findings of this study are openly available in Zenodo at http://doi.org/10.5281/zenodo.15024938. The code used for the analysis is available at: https://github.com/FranceCosta/isopeptide_bonds_global_survey.
