# Supplementary material for: A global survey of intramolecular isopeptide bonds
Source: Protein Sci. 2025 Nov 14;34(12):e70342. doi: 10.1002/pro.70342 (PMC12617251; doi:10.1002/pro.70342)
Supplement: Supplementary file 1 — Table S1. Prevalence of proximal waters <5 Å of intramolecular isopeptide bonds. Only one PDB entry was assessed per sequence‐identical domain. Table S2. Pfam domains detected with Isopeptor and total counts of domains from the AFDB. Domain assignment was executed as explained in the results section. Only domains detected at least 20 times are shown. False positives have been excluded. Table S3. A list of human‐binding pathogens/opportunistic pathogens that employ cell‐surface proteins containing intramolecular IPDs identified by Isopeptor. Table S4. Number and percentage of domains predicted to contain intramolecular isopeptide and disulfide bonds. Table S5. Number of domains predicted to contain both isopeptide and disulfide bonds and their % relative to the total number of IPDs for each phylum. Phyla with less than 100 counts are reported under “other.” Table S6. Sequences of synthetic DNA used in this study, and the source of pOPINF plasmid used for protein expression. pOPINF‐complementary sequences of primers are underlined in bold. Figure S1. (A) Average number of amino acids within 6 Å of the isopeptide bond centroids from PDB structures, per domain. Isopeptide bonds favor neighboring hydrophobic residues, especially aromatic ones: tyrosine in CnaA‐like domains and phenylalanine in CnaB‐like domains. (B) Scatterplot showing the distance from the isopeptide bond Nζ atoms to the closest aromatic ring and the angle between the aromatic ring and the isopeptide Cγ‐Nζ planes. The minimal distance to either the closest aromatic ring atom or to the aromatic ring center is reported. A minority of proximal aromatic rings (distance <3.8 Å) interact with the isopeptide Nζ atom with a conformation compatible with H‐bonding (interplanar angle around 90°; example a. PDB ID: 2X9W) while most of them engage in stacking interactions (planar angle <30° or planar angle >150°; example b. PDB ID 3GLE; Mitchell et al., 1994). Sequence redundancy was not removed in panel (B). Figure [file PRO-34-e70342-s001.docx]

## Supplementary tables

| **Intramolecular isopeptide bond type** | **Intramolecular IPDs with a water or ammonia molecule within 5 Å from the bond Oδ** |
| --- | --- |
| **CnaA-like** | 59% |
| **CnaB-like** | 54% |

**Table S1:** Prevalence of proximal waters <5 Å of intramolecular isopeptide bonds. Only one PDB entry was assessed per sequence-identical domain.

| **Pfam ID (accession code)** | **Pfam Clan ID (accession code)-isopeptide bond class type** | **Total number of domains present in the AFDB** | **Predicted to contain an intramolecular isopeptide bond** |
| --- | --- | --- | --- |
| Collagen_bind (PF05737) | Adhesin  (CL0204)  CnaA-like | 2,589 | 2,307 |
| GramPos_pilinBB (PF16569) |  | 2,226 | 2,044 |
| AgI_II_C2 (PF17998) |  | 876 | 684 |
| Antigen_C (PF16364) |  | 316 | 246 |
| Sgo0707_N2 (PF20623) |  | 274 | 171 |
| DUF7926 (PF25548) |  | 181 | 148 |
| DUF7929 (PF25551) |  | 162 | 123 |
| DUF7925 (PF25546) |  | 154 | 109 |
| DUF11 (PF01345) | E-set  (CL0159) CnaA-like | 25,913 | 11,756 |
| DUF7507 (PF24346) |  | 5,318 | 3,146 |
| DUF7619 (PF24595) |  | 1,981 | 1,000 |
| DUF7933 (PF25564) |  | 776 | 247 |
| GBS104-like_Ig (PF21426) |  | 425 | 235 |
| DUF7927 (PF25549) |  | 524 | 231 |
| DUF7617 (PF24593) |  | 78 | 73 |
| SpaA (PF17802) | Transthyretin  (CL0287)  CnaB-like | 27,202 | 16,929 |
| Cna_B (PF05738) |  | 6,407 | 5,013 |
| FctA (PF12892) |  | 3,002 | 2,170 |
| DUF5979 (PF19407) |  | 2,528 | 1,965 |
| GramPos_pilinD1 (PF16555) |  | 2,784 | 1,692 |
| DUF7601 (PF24547) |  | 818 | 668 |
| SpaA_4 (PF24514) |  | 567 | 378 |
| SpaA_2 (PF19403) |  | 1,259 | 246 |
| SpaA_3 (PF20674) |  | 622 | 196 |
| GramPos_pilinD3 (PF16570) |  | 165 | 137 |
| SdrD_B (PF17210) |  | 9,185 | 36 |

**Table S2**: Pfam domains detected with Isopeptor and total counts of domains from the AFDB. Domain assignment was executed as explained in the results section. Only domains detected at least 20 times are shown. False positives have been excluded.

| **Gram-positive pathogens** | **Gram-negative pathogens** |
| --- | --- |
| Abiotrophia defectiva | Acinetobacter baumannii |
| Actinomyces naeslundii | Acinetobacter haemolyticus |
| Actinomyces oris | Brucella abortus |
| Bacillus anthracis | Brucella melitensis |
| Bacillus cereus | Brucella suis |
| Bacillus thuringiensis | Catonella morbi |
| Clostridioides difficile | Legionella bozemanae |
| Clostridium botulinum | Porphyromonas gingivalis |
| Clostridium perfringens | Salmonella enterica |
| Clostridium tetani | Salmonella newport |
| Hathewaya histolytica (Clostridium histolyticum) | Salmonella typhimurium |
| Corynebacterium diphtheriae | Vibrio alginolyticus |
| Corynebacterium jeikeium | Vibrio parahaemolyticus |
| Corynebacterium striatum |  |
| Enterococcus faecalis |  |
| Enterococcus faecium |  |
| Listeria grayi |  |
| Listeria ivanovii |  |
| Listeria monocytogenes |  |
| Staphylococcus aureus |  |
| Streptococcus agalactiae |  |
| Streptococcus anginosus |  |
| Streptococcus downei |  |
| Streptococcus dysgalactiae |  |
| Streptococcus gordonii |  |
| Streptococcus mitis |  |
| Streptococcus mutans |  |
| Streptococcus oralis |  |
| Streptococcus pneumoniae |  |
| Streptococcus pyogenes |  |
| Streptococcus sanguinis |  |

**Table S3:** A list of human-binding pathogens/opportunistic pathogens that employ cell-surface proteins containing intramolecular IPDs identified by Isopeptor.

| **Pfam ID (accession code)** | **Pfam Clan ID (accession code)-isopeptide bond class type** | **Number of domains with predicted isopeptide and disulfide bonds** | **% of IPDs with disulfide bonds** |
| --- | --- | --- | --- |
| SpaA (PF17802) | Transthyretin  (CL0287)  CnaB-like | 1,507 | 8.9 |
| DUF5979 (PF19407) |  | 783 | 39.8 |
| SpaA_4 (PF24514) |  | 376 | 99.5 |
| SpaA_2 (PF19403) |  | 235 | 95.5 |
| GramPos_pilinD1 (PF16555) |  | 199 | 11.8 |
| SpaA_3 (PF20674) |  | 195 | 99.5 |
| FctA (PF12892) |  | 109 | 5.0 |
| Cna_B (PF05738) |  | 22 | 0.4 |
| SdrD_B (PF17210) |  | 5 | 13.9 |
| DUF7601 (PF24547) |  | 3 | 0.4 |
| DUF11 (PF01345) | E-set  (CL0159) CnaA-like | 3,497 | 29.7 |
| DUF7507 (PF24346) |  | 1,099 | 34.9 |
| DUF7933 (PF25564) |  | 240 | 97.2 |
| DUF7927 (PF25549) |  | 179 | 77.5 |
| DUF7617 (PF24593) |  | 71 | 97.3 |
| DUF7619 (PF24595) |  | 11 | 1.1 |
| Sgo0707_N2 (PF20623) | Adhesin  (CL0204)  CnaA-like | 13 | 7.6 |
| DUF7926 (PF25548) |  | 8 | 5.4 |
| GramPos_pilinBB (PF16569) |  | 7 | 0.3 |
| Collagen_bind (PF05737) |  | 3 | 0.1 |

**Table S4**: Number and percentage of domains predicted to contain intramolecular isopeptide and disulfide bonds.

| **Kingdom** | **Phylum** | **Number of domains with predicted isopeptide and disulfide bonds** | **% of IPDs with disulfide bonds** |
| --- | --- | --- | --- |
| Bacteria | Actinomycetota | 4,757 | 41.3 |
| Bacteria | Pseudomonadota | 1,384 | 58.2 |
| Bacteria | Chloroflexota | 549 | 42.7 |
| Bacteria | Acidobacteriota | 263 | 79.2 |
| Bacteria | Deinococcota | 101 | 25.2 |
| Bacteria | Other | 936 | 2.8 |
| Archaea | Euryarchaeota | 173 | 20.8 |
| Archaea | Other | 158 | 32.4 |

**Table S5**: Number of domains predicted to contain both isopeptide and disulfide bonds and their % relative to the total number of IPDs for each phylum. Phyla with less than 100 counts are reported under “other”.

| **Gene constructs (codon optimised for Escherichia coli)** | |
| --- | --- |
| **CLIPPER_WT_** | AAACCGACCGTTGATGTTGTTAAAACCACCACCGCAACCACCGCCAAAGTTGGTGATACCATTGATTATACCGTTAAAGTTACCGTTGCAAATAGCCAGACCACCGATGCACTGACCCTGAATGATACCTTAGGTCAGGGTCTGAGCTTTGTTAGCGGCACCGCACCGACCGGTTGGACCCTGACAGGTAATGGTCAGGCAATTAACATTGCAGCACCGAAAGGTCAGATTCCGGGTACATATAATCTGACGTATAAAGTTCTGGTTGGCACCGATGCCGTTAATAATGTTGTGAATAAAGTGACCGCAAGCGGTGGTGATAAACCGAGCTGCACCACCTGTACCACCACCACACCGGTTACC |
| **CLIPPER_K715A_** | AAACCGACCGTTGATGTTGTTGCAACCACCACCGCAACCACCGCCAAAGTTGGTGATACCATTGATTATACCGTTAAAGTTACCGTTGCAAATAGCCAGACCACCGATGCACTGACCCTGAATGATACCTTAGGTCAGGGTCTGAGCTTTGTTAGCGGCACCGCACCGACCGGTTGGACCCTGACAGGTAATGGTCAGGCAATTAACATTGCAGCACCGAAAGGTCAGATTCCGGGTACATATAATCTGACGTATAAAGTTCTGGTTGGCACCGATGCCGTTAATAATGTTGTTAATAAAGTGACCGCAAGCGGTGGTGATAAACCGAGCTGCACCACCTGTACCACCACCACACCGGTTACC |
| **Primers used in this study** | |
| **CLIPPER constructs Forward primer** | **AAGTTCTGTTTCAGGGCCCG**AAACCGACCGTTGATGTTGTT |
| **CLIPPER constructs Reverse primer** | **ATGGTCTAGAAAGCTTTA**GGTAACCGGTGTGGTGGTG |
| **Plasmid used in this study** | |
| **pOPINF** | Ampicillin-resistant expression vector for recombinant proteins, incorporating an N-terminal cleavable hexa-histidine tag (MAHHHHHHSSGLEVLFQGP, Berrow et al. 2007) |

**Table S6:** Sequences of synthetic DNA used in this study, and the source of pOPINF plasmid used for protein expression. pOPINF-complementary sequences of primers are underlined in bold.

##

## Supplementary figures


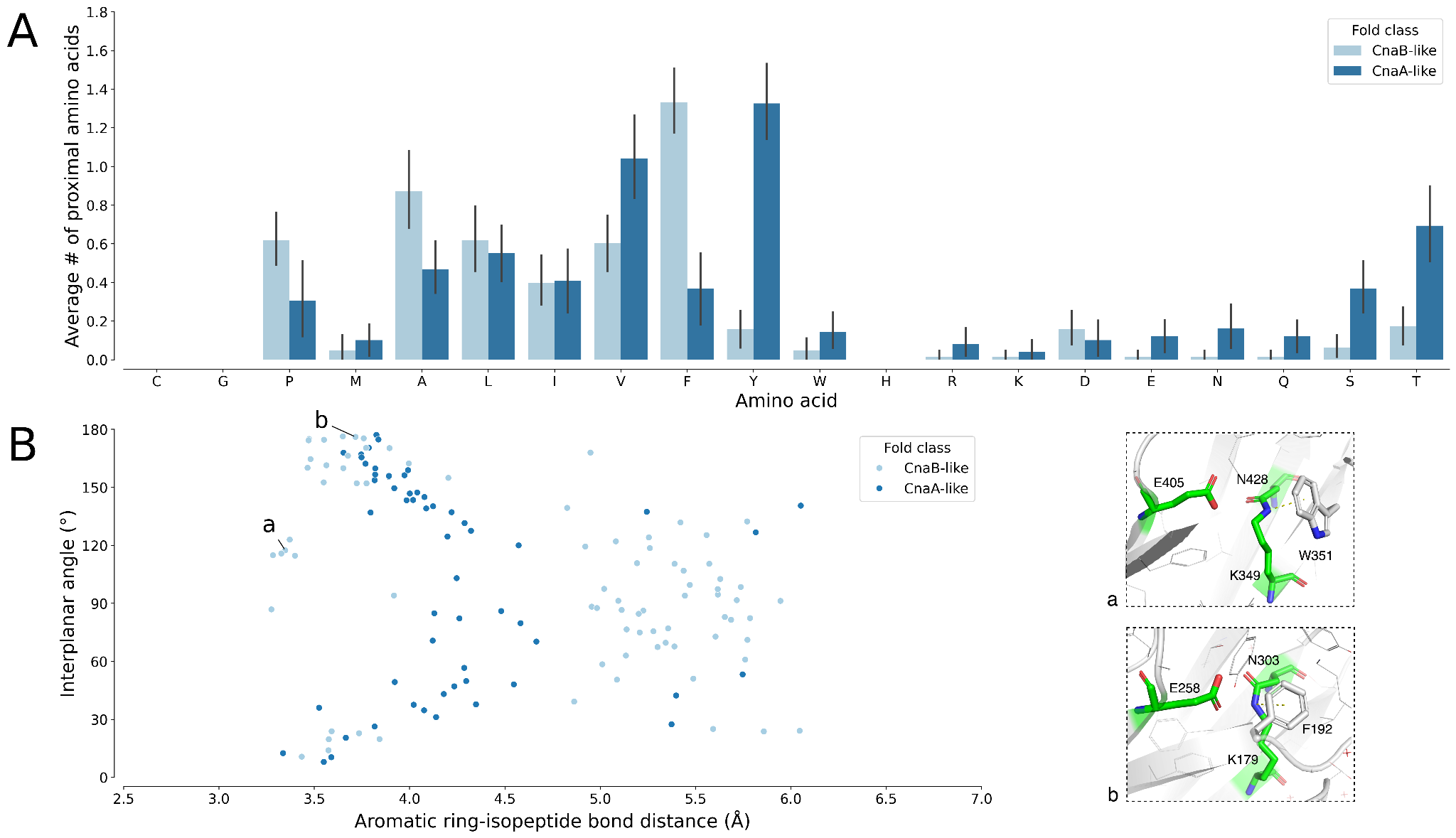


**Figure S1**: A. Average number of amino acids within 6Å of the isopeptide bond centroids from PDB structures, per domain. Isopeptide bonds favour neighbouring hydrophobic residues, especially aromatic ones: tyrosine in CnaA-like domains and phenylalanine in CnaB-like domains. B. Scatterplot showing the distance from the isopeptide bond Nζ atoms to the closest aromatic ring and the angle between the aromatic ring and the isopeptide Cγ-Nζ planes. The minimal distance to either the closest aromatic ring atom or to the aromatic ring centre is reported. A minority of proximal aromatic rings (distance < 3.8 Å) interact with the isopeptide Nζ atom with a conformation compatible with H-bonding (interplanar angle around 90°; example a. PDB ID: 2X9W) while most of them engage in stacking interactions (planar angle < 30° or planar angle > 150°; example b. PDB ID 3GLE; Mitchell et al. 1994). Sequence redundancy was not removed in panel B.

**
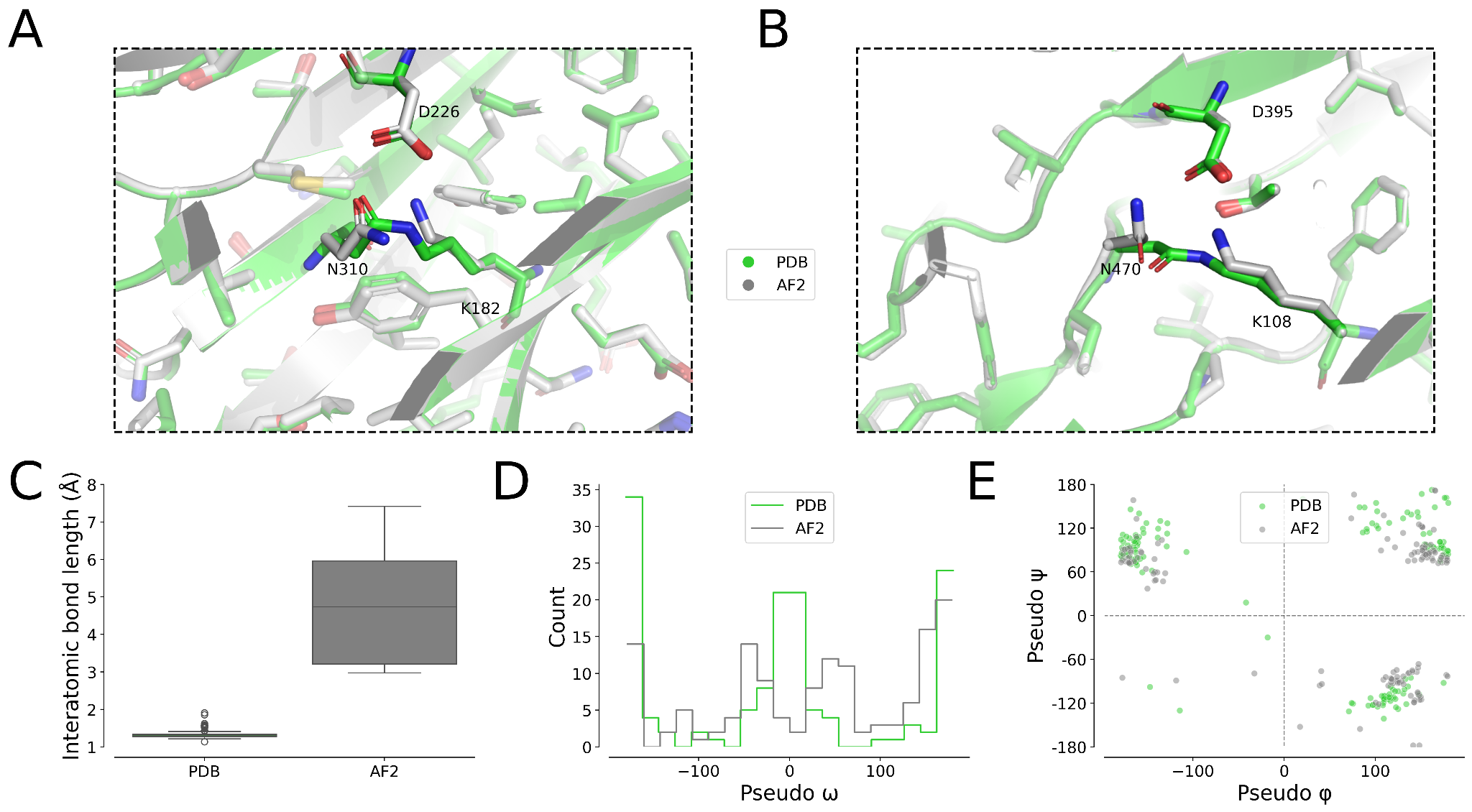
**

**Figure S2**: A. An example in which the intramolecular isopeptide bond modelled by AlphaFold2 resembles the bond of the PDB structure (PDB ID: 5XCB). B. An example in which the intramolecular isopeptide bond modelled by AlphaFold2 does not resemble the one found in the PDB structure (PDB ID: 6M3Y). For panels A and B, both intramolecular isopeptide bonds are confidently predicted by Isopeptor (probability 0.89 and 0.73, respectively). The average pLDDT of isopeptide bond side chains in the AlphaFold2 models is >90 (see Figure S5A). C. Isopeptide bond length distributions as calculated between the atoms that form the covalent link (Lys_Nζ_ and Asp/Asn_Cɣ_). D. Dihedral pseudo ω angle distributions. E. pseudo ψ and φ angle distributions. Pseudo dihedral angles were calculated as described in Costa et al*.* 2025. The difference in distances between asparagine/aspartate and lysine side chains in AF2 models likely reflects an attempt to prevent local clashes. This is also reflected in the distribution of pseudo ω angles (in which the *cis* conformation is unfavourable in AlphaFold2 models). Sequence redundancy removal was not applied in this figure.


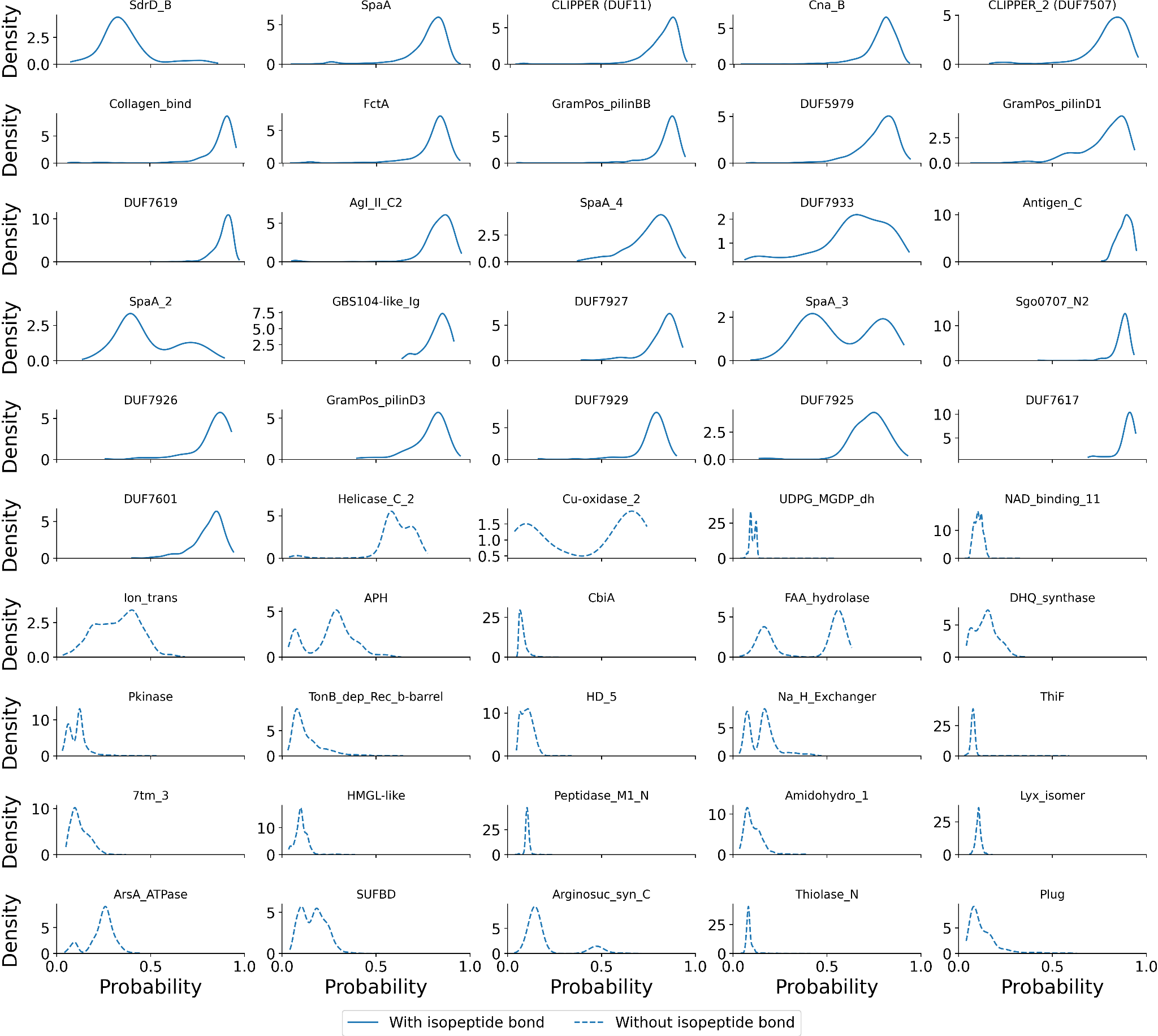
**Figure S3**: Distribution of Isopeptor probabilities for domains which contain an intramolecular isopeptide bond and for domains unlikely to contain an isopeptide bond. Intramolecular IPDs exhibit a higher Isopeptor probability score (above the threshold of 0.65), indicating that Isopeptor is detecting isopeptide bonds with high recall. There are a few exceptions to this: SpaA_2, SpaA_3 and SdrD_B domains have a substantial fraction of isopeptide bonds predicted with low confidence, likely due to a lack of experimental templates closely matching their isopeptide bond geometries. Helicase_C_2 and Cu-oxidase_2 domains, which have a substantial fraction of isopeptide bond signatures above the probability threshold of 0.65, were determined to be false positives.


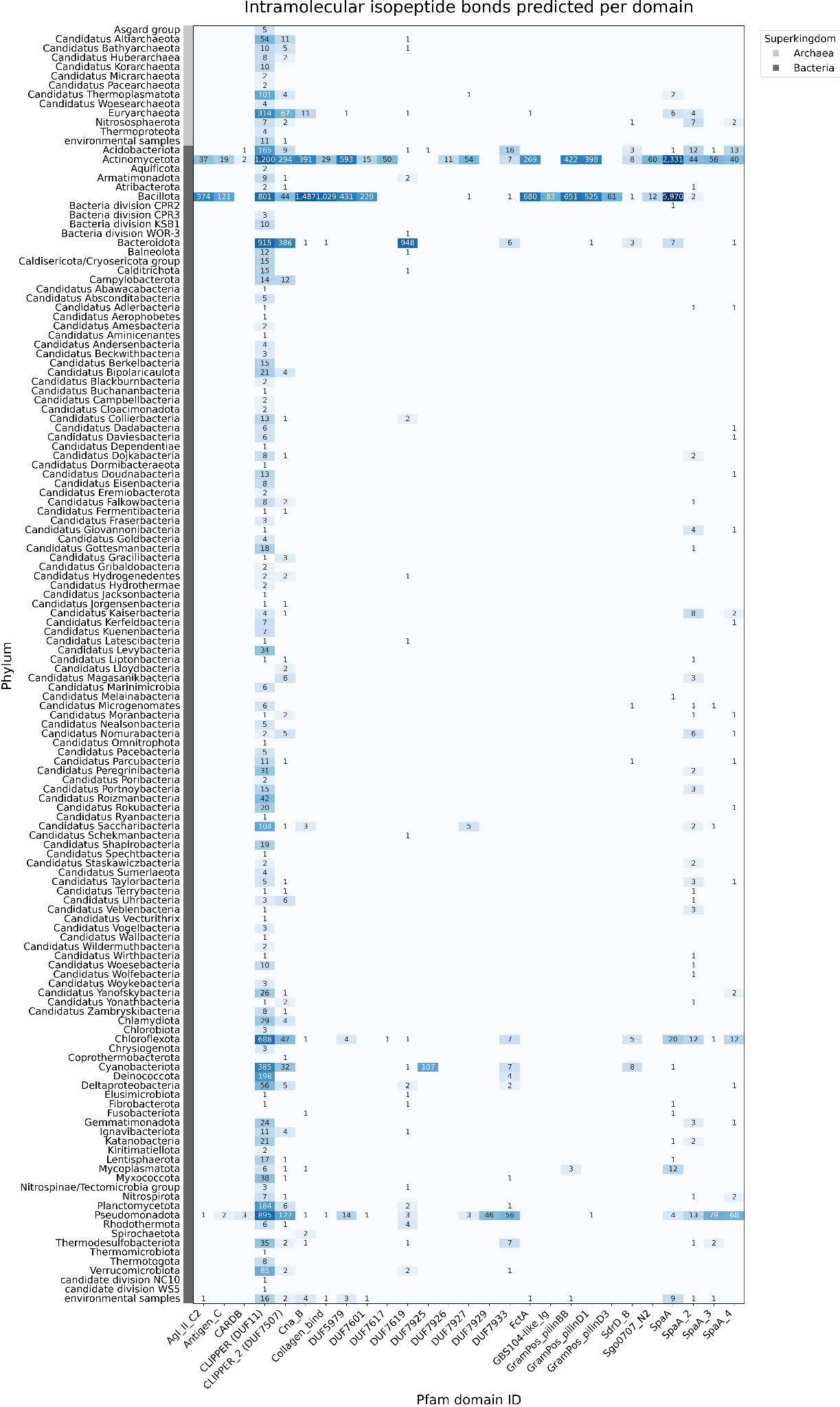


**Figure S4**: Distribution of intramolecular IPDs per phylum and superkingdom, detected with Isopeptor.

**
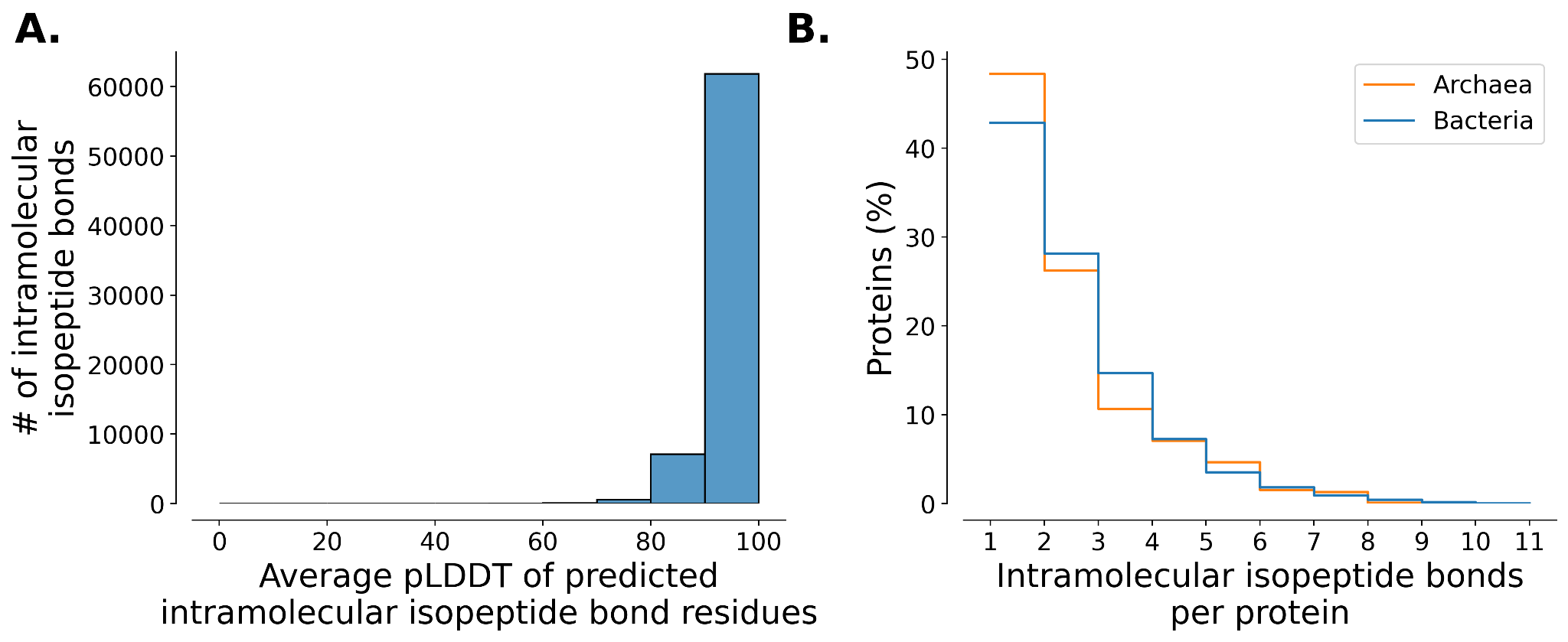
**

**Figure S5**: A. Distribution of pLDDT values for predicted intramolecular isopeptide bond residues across the AFDB. B. Intramolecular isopeptide bonds detected per protein. 57% and 52% of bacterial and archaeal proteins detected by Isopeptor contain more than one isopeptide bond, respectively.


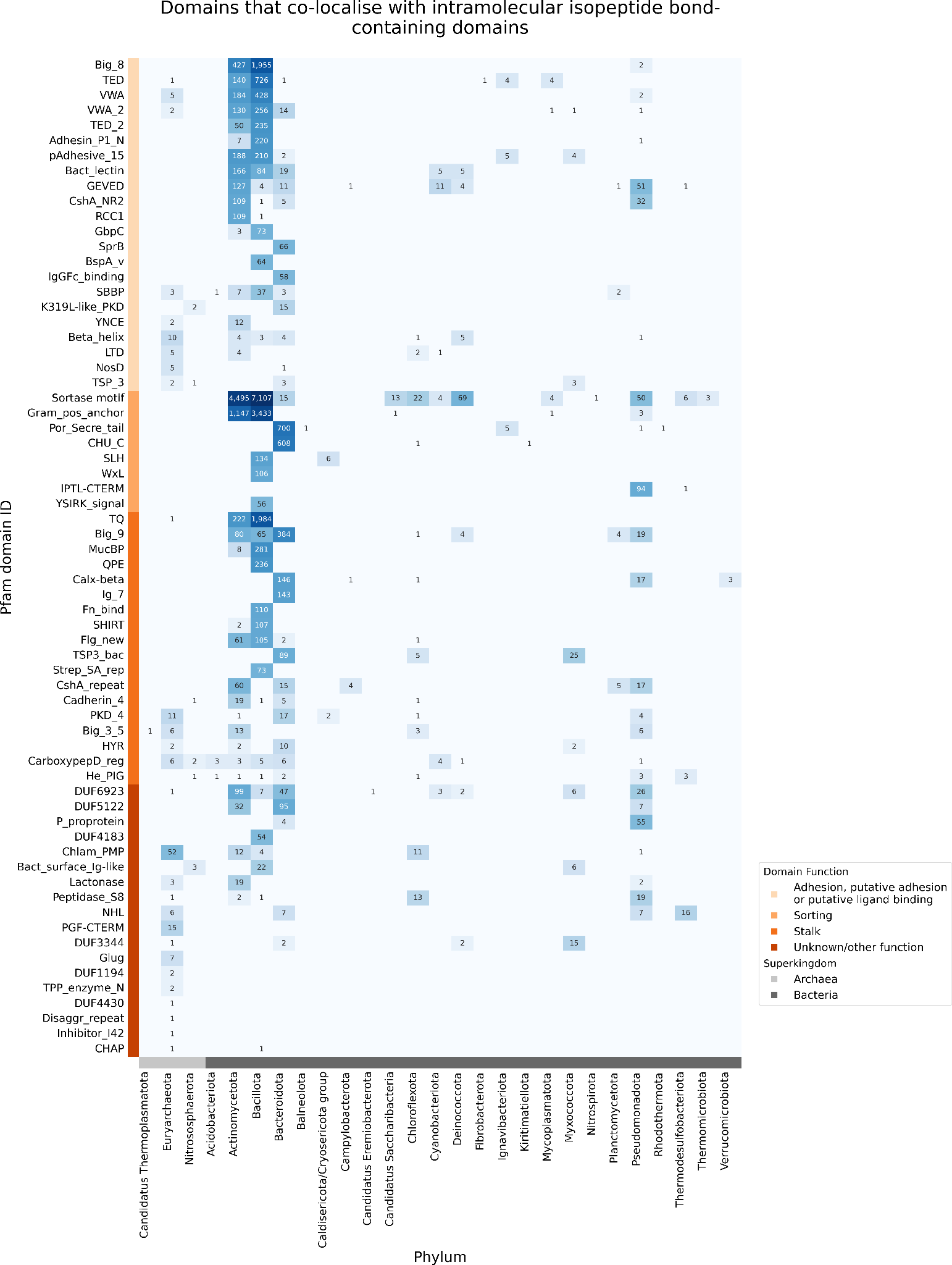
**Figure S6:** Domains co-occurring with intramolecular IPDs, divided into functional classes and phylum. The most common functional classes are adhesion, sorting and stalk.

**
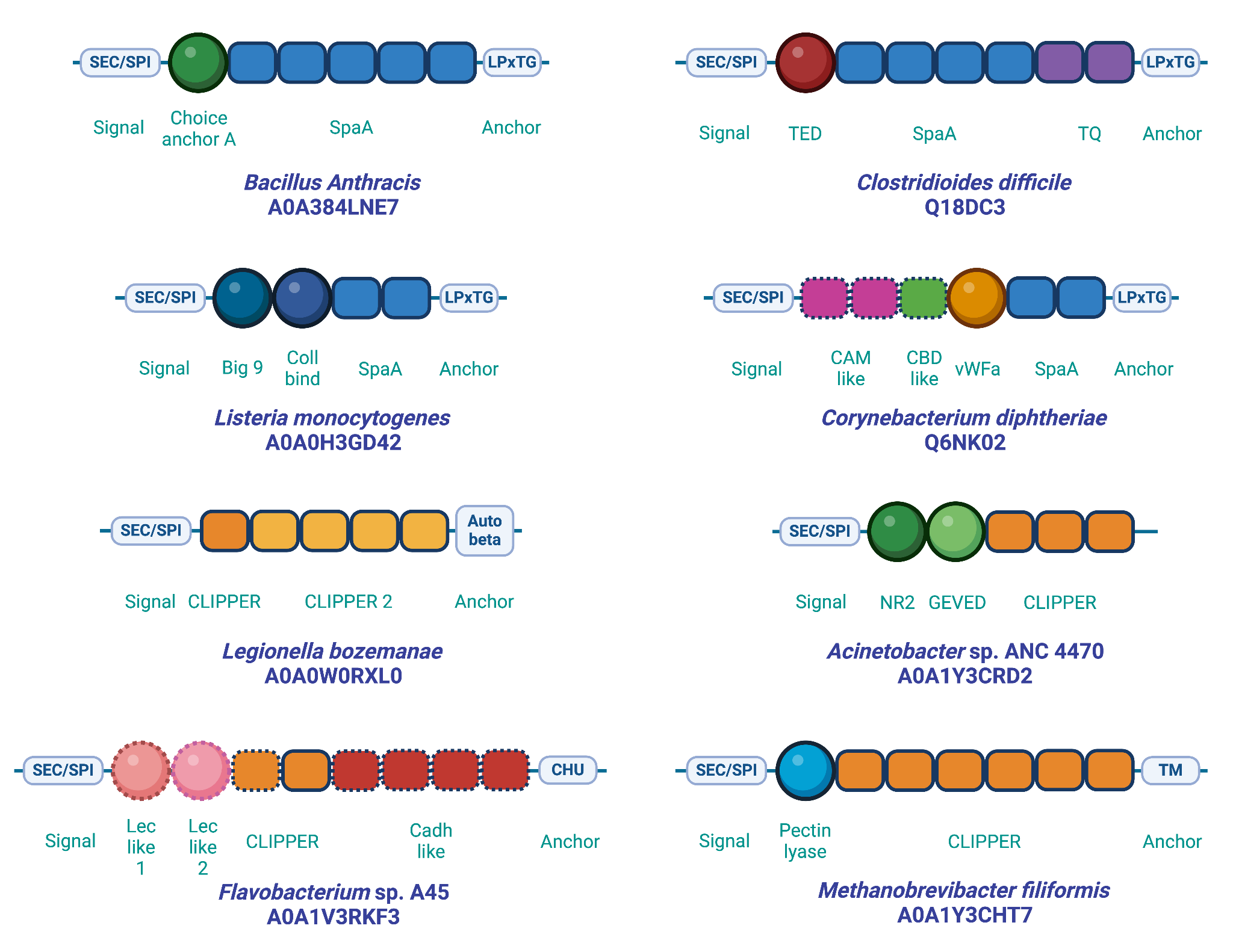
**

**Figure S7.** A selection of AFDB proteins predicted to contain intramolecular isopeptide domains, identified by Isopeptor. Adhesive domains are depicted as circles, stalk domains are depicted as rounded boxes, and anchor motifs/domains and signal peptides are depicted in light blue boxes at the C and N termini, respectively. Domains are colour coded by domain identity. Dotted outlines indicate domains that could not be correlated to Pfam domain families using sequence information, but that exhibit predicted structures that are similar to other domains of known function. Domain families are listed below each domain or domain repeat region. TED = Thioester Domain, TQ = T-Q ester bond domain, Coll bind = Collagen binding domain, CAM like = Cell adhesion module-like domain, CBD like = Carbohydrate binding-like domain, Lec like = Lectin-like domain, Cadh like = Cadherin-like domain.

**
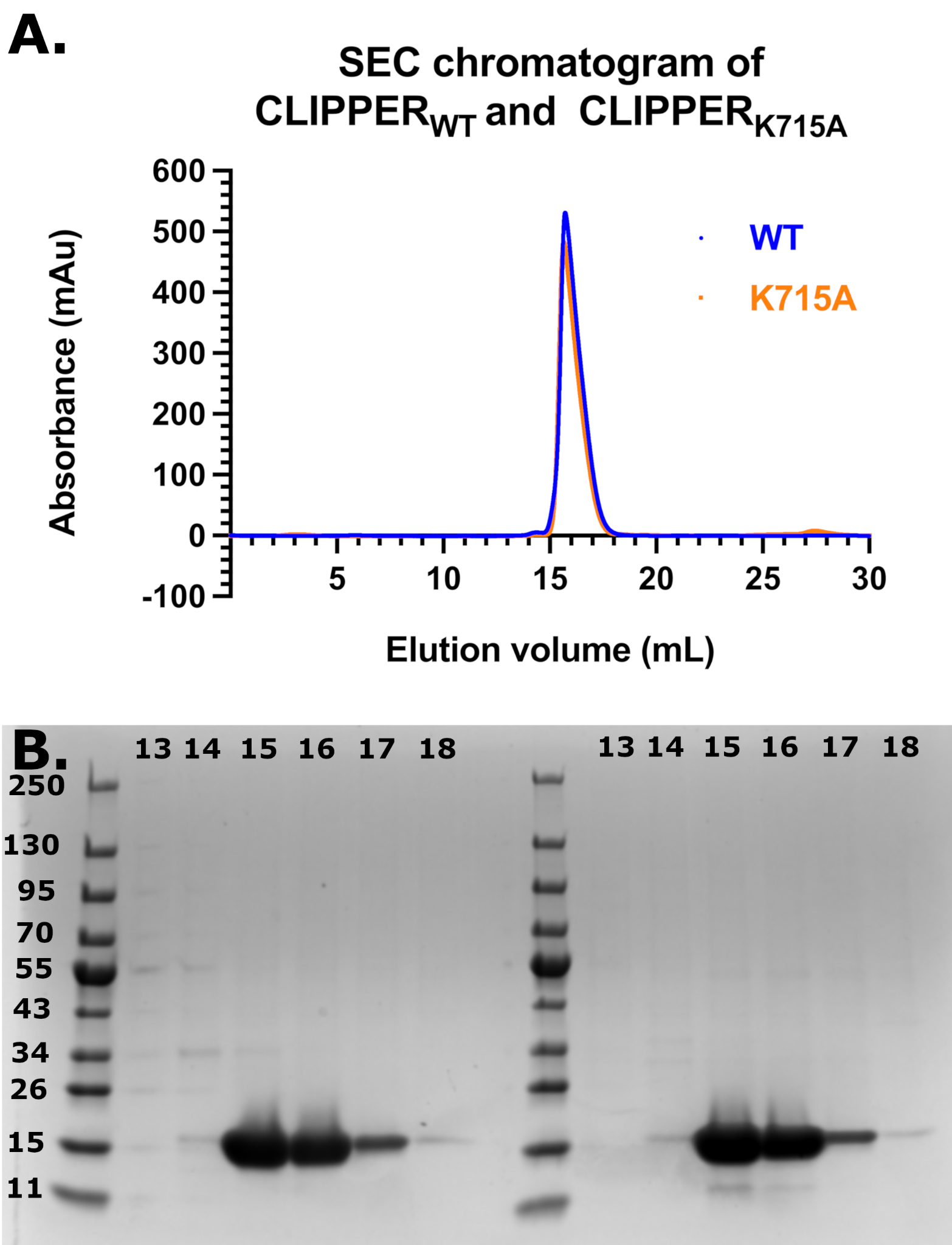
**

**Figure S8.** A. SEC chromatograms of CLIPPER_WT_ and CLIPPER_K715A_, demonstrating one primary peak per polypeptide indicative of a monomer. B. SDS-PAGE analysis of eluted SEC fractions of CLIPPER_WT_ (left) and CLIPPER_K715A_ (right).
